# Long-range epigenetic regulation is conferred by genetic variation located at thousands of independent loci

**DOI:** 10.1038/ncomms7326

**Published:** 2015-02-26

**Authors:** Mathieu Lemire, Syed H.E. Zaidi, Maria Ban, Bing Ge, Dylan Aïssi, Marine Germain, Irfahan Kassam, Mike Wang, Brent W. Zanke, France Gagnon, Pierre-Emmanuel Morange, David-Alexandre Trégouët, Philip S. Wells, Stephen Sawcer, Steven Gallinger, Tomi Pastinen, Thomas J. Hudson

**Affiliations:** 1 Ontario Institute for Cancer Research, Toronto, Ontario, Canada M5G 0A3; 2 Department of Clinical Neurosciences, University of Cambridge, Cambridge Biomedical Campus, Hills Road, Cambridge CB2 0QQ, UK; 3 McGill University and Genome Québec Innovation Centre, Montréal, Québec, Canada H3A 0G1; 4 INSERM, UMR-S 1166, Paris F-75013, France; 5 Sorbonne Universités, UPMC Univ Paris 06, UMR_S 1166, Team Genomics and Pathophysiology of Cardiovascular Diseases, Paris F-75013, France; 6 ICAN Institute for Cardiometabolism and Nutrition, Paris F-75013, France; 7 Dalla Lana School of Public Health, University of Toronto, College Street, Toronto, Ontario, Canada M5T 3M7; 8 Department of Medicine, University of Ottawa, Ottawa Hospital Research Institute, Ottawa, Ontario, Canada K1H 8L6; 9 Laboratory of Haematology, La Timone Hospital, Marseille F-13385, France; 10 INSERM, UMR_S 1062, Nutrition Obesity and Risk of Thrombosis, Marseille F-13385, France; 11 Aix-Marseille University, UMR_S 1062, Nutrition Obesity and Risk of Thrombosis, Marseille F-13385, France; 12 Samuel Lunenfeld Research Institute, Toronto, Ontario, Canada M5S 1X5; 13 Division of General Surgery, Toronto General Hospital, Toronto, Ontario, Canada M5G 2C4; 14 Department of Medical Biophysics, University of Toronto, Toronto, Ontario, Canada M5S 1A1; 15 Department of Molecular Genetics, University of Toronto, Toronto, Ontario, Canada M5S 1A1

## Abstract

The interplay between genetic and epigenetic variation is only partially understood. One form of epigenetic variation is methylation at CpG sites, which can be measured as methylation quantitative trait loci (meQTL). Here we report that in a panel of lymphocytes from 1,748 individuals, methylation levels at 1,919 CpG sites are correlated with at least one distal (*trans*) single-nucleotide polymorphism (SNP) (*P*<3.2 × 10^−13^; FDR<5%). These *trans*-meQTLs include 1,657 SNP–CpG pairs from different chromosomes and 262 pairs from the same chromosome that are >1 Mb apart. Over 90% of these pairs are replicated (FDR<5%) in at least one of two independent data sets. Genomic loci harbouring *trans*-meQTLs are significantly enriched (*P*<0.001) for long non-coding transcripts (2.2-fold), known epigenetic regulators (2.3-fold), piwi-interacting RNA clusters (3.6-fold) and curated transcription factors (4.1-fold), including zinc-finger proteins (8.75-fold). Long-range epigenetic networks uncovered by this approach may be relevant to normal and disease states.

## Abstract (web summary)

There is a functional link between SNPs and epigenetic variations when they are in close range, but the long-range effect is unclear. Here, by analysing methylation quantitative trait loci, the authors demonstrate that methylation levels at CpG sites in lymphocytes are correlated with distal SNPs.

## Graphical abstract

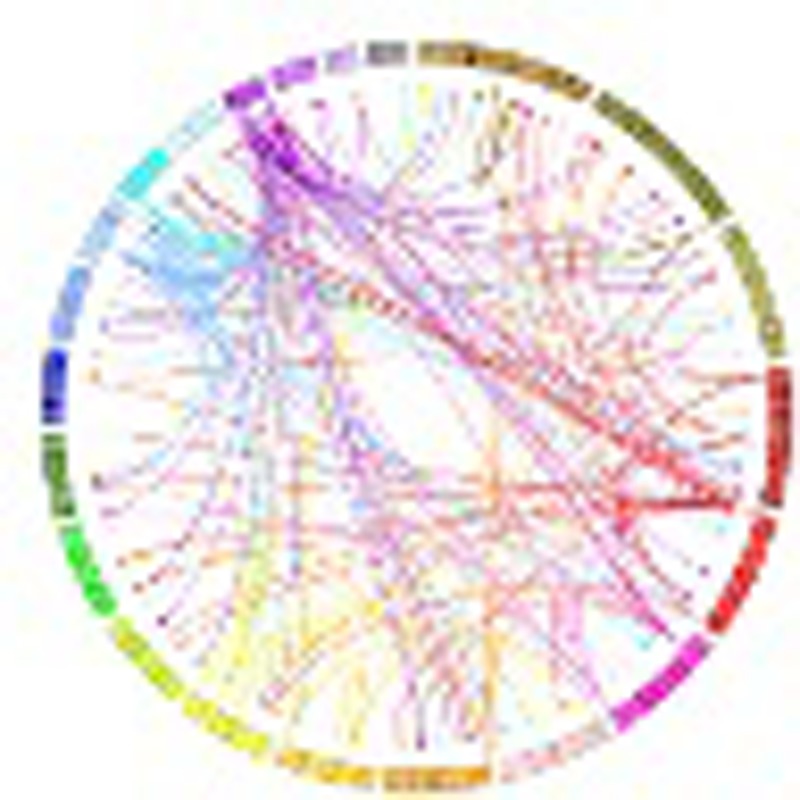

Characterizing the relationships between genetic variants and functional elements of the genome is needed to advance our understanding of phenotypic diversity and characterize how genetic variation can perturb cell function and affect disease predisposition. Although cells contain a myriad of biomolecules that can potentially be assayed across large numbers of genetically characterized samples, analyses of nucleic elements such as transcript expression and CpG methylation are ideally suited for genome-wide comparisons with currently available technologies. In general, the detection of association between nearby (*cis*) genetic variants and functional elements has been easier to detect and validate than distal (*trans*) relationships, because of the multiple testing burden that arises when *trans*-associations are explored.

Expression quantitative trait loci (eQTL) studies in the past decade have demonstrated that gene transcript levels in a cell are frequently associated with nearby genetic variants (*cis*-eQTLs), which in turn are enriched for single-nucleotide polymorphisms (SNPs) associated with disease^1^,^2^. In parallel, mechanistic links between the disease-associated variants and the biology of the disease governed by local gene expression alterations are emerging^3^. The detection of eQTLs spanning long distances (*trans*-eQTLs) has been challenging, particularly due to the multiple testing burden that occurs when genome-wide sets of variants are compared with genome-wide sets of genes. To address this limitation, Westra *et al*.^4^ performed an eQTL meta-analysis in 5,311 peripheral blood samples. They furthermore reduced the number of variants to test for *trans*-eQTLs to 4,542 SNPs that have been implicated in complex traits by genome-wide association studies. This allowed the detection and replication of *trans*-eQTLs for 233 disease-associated SNPs (at 103 independent loci), and provided insight into the pathogenesis of disease for selected variants identified in their screen.

An alternate approach to mapping regulatory relationships between genetic variants and distal genes is by correlating genetic variants with genome-wide epigenomic profiles. Notwithstanding rapid developments in epigenome-mapping methods that can explore a large number of chromatin modifications, the only approach that can screen thousands of samples needed to detect *trans*-acting relationships in a genome-wide fashion is an array-based multiplex assay that interrogates a multitude of methylation sites in parallel. A first-generation array that measured methylation levels at 22,290 CpG dinucleotides in 77 lymphoblastoid cell lines was used to detect associations between genome-wide SNPs and CpG methylation^5^. After applying a genome-wide false discovery rate (FDR) of 10% (*P*=2.1 × 10^−10^), they detected 27 putative *cis*-association signals, most of which involved SNPs and CpG sites located within 50 kb of each other and 10 *trans*-association signals. Despite the limitations in the sample size used in this study, these preliminary studies demonstrated the potential to map *trans*-regulatory relationships between genetic variants and distal epigenetic elements that may affect gene regulation and complex disease phenotypes.

Here, we report a methylation quantitative trait loci (meQTL) study involving an analysis of methylation profiles for 380,189 CpGs and genotypes for 410,203 SNPs determined in lymphocytes from 898 patients with colon cancer and 850 controls, and subsequent replication in two independent data sets totalling 577 samples. To characterize the *trans*-meQTL loci that are statistically significant, we determined whether these loci are enriched in protein-coding and RNA-coding genes that could mediate the *trans*-acting effects. We explored *cis*- and *trans*-eQTLs correlating with meQTL loci in transcriptomic data sets derived from 137 CD4 and 137 CD8 lymphocytes. The analyses demonstrate an abundance of genetic loci that are associated with distal CpG methylation, a diversity of regulatory mechanisms that confer this role and networks of coordinated genes that are linked to biological and/or disease processes.

## Results

### Description of the data set

In this study, genome-wide SNP data and CpG methylation profiles detected using the Infinium HumanMethylation450 BeadChips were obtained from DNA extracted from lymphocytes of 898 patients with colon cancer and 850 controls (after sample exclusions, see Methods) enrolled in the Ontario Familial Colon Cancer Registry (OFCCR)^6^,^7^. We normalized the methylation data sets and identified and flagged sites with more than 1% missing data (*n*=13,032), calculated after sample exclusions. We further identified CpG sites that are polymorphic irrespective of the minor allele frequency (MAF), sites for which at least one SNP with a MAF above 5% resides anywhere else underneath the probe sequence (69,974 sites derived from ref. 8) and sites that cross-hybridize with >90% identity to multiple regions (30,436 sites). These overlapping counts amount to 93,675 unique sites that were discarded. We retained the methylation values (also known as the *β*-values) at 380,189 autosomal CpG sites, as well as genotypes for 410,203 SNPs for further analyses. The OFCCR methylation and SNP data were deposited in dbGaP under the accession number phs000779.v1.p1.

For the data analyses that follow, we classify a SNP–CpG association as proximal if the SNP and the CpG site are separated by no more than 1 Mbp on the same homologous chromosome and as distal otherwise. Over 103 million SNP–CpG pairs were proximal candidates, and over 155 billion pairs were distal candidates. For each CpG site, we looked for SNPs that are associated with methylation levels in the 1,748 DNA samples; we note that we only combined cases and controls after performing separate analyses, which revealed over 80% overlap in significant results. The quantitative trait analyses were adjusted for sex, age, blood cell type surrogate (see Methods and Supplementary Fig. 1), batch (plate) and position of the sample on its array to get the significance and the proportion of the methylation variance explained by a SNP. We retained SNP–CpG associations consistent with an FDR (ref. 9) <5%, and report the effect size as the proportion *R*^2^ of the CpG methylation variance that is explained by the SNP, among the variance not already explained by sex, age, blood cell type surrogate, batch and array position.

### Proximal SNP–CpG associations

Owing to a rich body of literature^5^,^10^,^11^,^12^,^13^,^14^,^15^,^16^,^17^,^18^,^19^, we present proximal SNP–CpG association results for completeness, but focus the rest of the study on distal (long range) associations.

Table 1 illustrates the distribution of the 52,708 CpG sites significantly associated with a proximal SNP (see also Supplementary Data 1), at an FDR <5%. At this FDR threshold, these CpG sites have at least 2.2% of their variance explained by at least one proximal SNP, with corresponding (unadjusted) significance level *P*_LRT_<4.8 × 10^−10^ derived from a likelihood ratio test (LRT). Following up these 52,708 sites by adjusting for additional covariates to account for hidden confounders (the top three principal components, that explain 92.5% of the variance) does not substantially alter this list: except for 313, all remain significant at FDR <5%; the 313 sites that failed this threshold nevertheless all have *P*_LRT_<1.8 × 10^−6^, with the large majority having *P*_LRT_<10^−8^. The number of significant sites decreases with increasing variance explained; at 75% variance explained (*R*^2^>0.75), the number of significant sites drops to 338. Table 1 further breaks down the set of CpG sites based on their variability, stratified in quintiles. As the methylation variability of the CpG site increases, the relative proportion of sites correlating with a proximal SNP increases, as well as the proportion of the variance that they explain.

### Distal SNP–CpG associations

We identified 1,919 CpG sites for which inter-individual methylation variations are significantly correlated with at least one distal SNP: 1,657 SNP–CpG pairs from non-homologous chromosomes and 262 SNP–CpG distant pairs (>1 Mb) located on the same homologous chromosome (Supplementary Data 2). These counts excluded CpG sites for which a proximal SNP was already identified. At an FDR <5%, these distal SNPs explain at least 3.1% of the variance of their companion CpG site, at a significance level *P*_LRT_<3.2 × 10^−13^. Following up these 1,919 sites by adjusting the analysis for additional covariates consisting of the top three principal components does not substantially alter this list: except for 33, all remain significant at FDR <5%; the 33 sites that failed to pass this threshold nevertheless all have *P*_LRT_<1.5 × 10^−10^. The case–control status is not a systematic or substantial confounder: at an FDR <5%, only 64 of the 1,919 sites (3.3%) display significant differences between cases and controls, no more than what is expected by chance alone. The very large majority (83.6%, or 1,605 pairs) of the 1,919 distal pairs are replicated in one (496 pairs) or both (1,109 pairs) replication sets (in MARTHA and/or F5L, both totalling 577 whole-blood samples) at FDR <5% (Supplementary Data 2), while replication of 8.5% of the pairs (165) was not attempted in both data sets due to poor quality of the intensity signals at the CpG sites or poor imputation quality (*R*^2^<0.3) of the SNPs; ignoring the pairs that were not successfully attempted, the replication rate is thus 91.5%.

Similar to proximal pairs, where sites that showed greater inter-individual variability are more likely to be associated with a proximal SNP, the proportion of sites associated with a distal SNP also increases with the site’s variability, but the increase is not as steep (Table 2).

**Figure 1:**
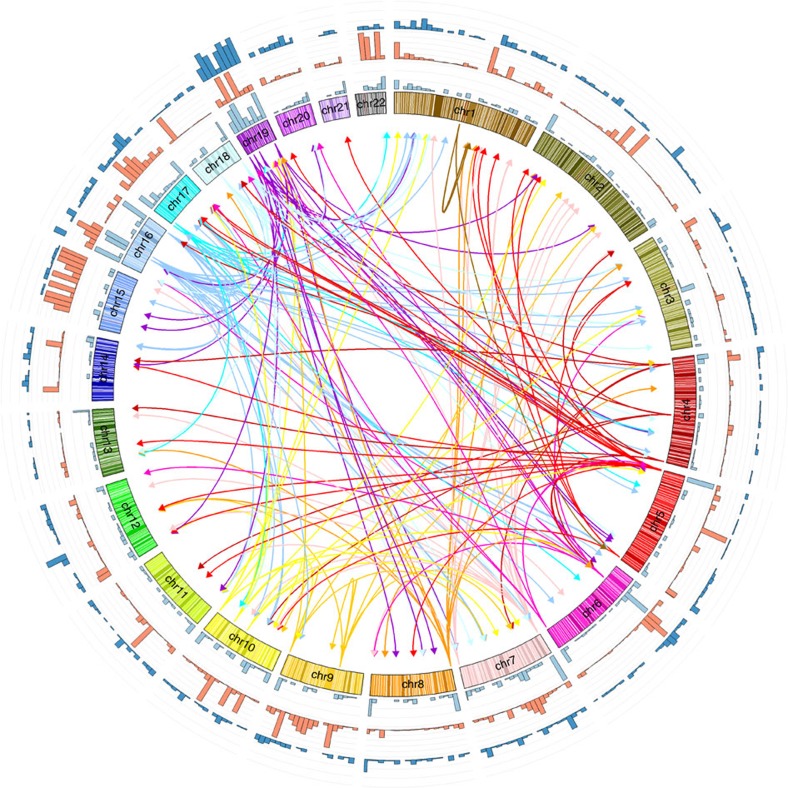
Enrichment of features in regions harbouring SNPs involved in distal SNP–CpG associations. Outer histograms: number of SNPs involved in distal SNP–CpG associations (light blue), calculated in 7.5 Mb bins; number of piRNA sequences (orange); number of transcription factors (dark blue). Inner links: SNP regions associated with four or more CpG sites. Arrows are pointing from SNPs to the CpG sites they are associated with, and are coloured according to the chromosomes where the SNPs reside.

The 1,387 unique SNPs (Supplementary Data 2) involved in the 1,919 significant SNP–CpG distal associations, as well as SNPs in linkage disequilibrium (LD) (*R*^2^>0.5) with them, define the boundaries of a total of 1,074 non-overlapping genomic regions totalling 109.4 Mb. Figure 1 and Supplementary Fig. 2 illustrate the genomic landscape of meQTL loci. The regions surrounding the SNPs harbour 2,167 RefSeq genes (1,798 coding and 369 non-coding transcripts, including 314 long (>100 bp) non-coding transcripts). We observe enrichment for coding (1.7-fold), non-coding transcripts (2.0-fold) and long non-coding transcripts (2.2-fold). We compiled a list of 190 autosomal genes involved in epigenetic processes^20^ (http://www.sabiosciences.com/rt_pcr_product/HTML/PAHS-085A.html; http://www.sabiosciences.com/rt_pcr_product/HTML/PAHS-086A.html) (Supplementary Data 3) and find 25 in our regions (13.2%, 2.3-fold enrichment). Of the 1,225 manually curated, high-confidence autosomal genomic loci that encode transcription factors (TFs)^21^, 244 are found in the neighbourhood of these SNPs (19.9%, 4.1-fold enrichment). Of the 388 manually curated TF that are zinc-finger proteins (ZNFs), 155 are in our regions (39.9%, 8.75-fold enrichment). Also noteworthy in these regions are the presence of piRNA (piwi-interacting RNA; Genbank accession numbers (with DQ prefix) enumerated in ref. 22): 3,072 piRNA sequences are detected, typically in clusters, near these SNPs; this corresponds to a 3.6-fold enrichment compared with random regions of the genome. Repetitive elements such as short interspersed nucleotide elements (1.2-fold), and more specifically of the Alu family (1.5-fold), were also slightly enriched. Note that for all of the above features, the observed number of features per Mb exceeds the corresponding number of features in randomly selected regions in all 1,000 replicates, effectively providing point estimates of significance of *P*<0.001 (estimated from proportions) for each of the above absolute fold enrichments. We also observe that 195 (18.1%) of the 1,074 non-overlapping genomic regions harbouring genetic variants associated with meQTLs are located towards the subtelomeric regions of the chromosomes (within 2 Mbp of the chromosome’s ends), as well as several small genomic regions containing one or more SNPs associated with multiple CpG sites.

Genes in the SNP LD-defined neighbourhoods and genes annotated to the CpG sites they are associated with were projected into functional interaction (FI) networks^23^ (a high-throughput network of protein–protein interactions) to evaluate which pairs display protein–protein interactions, or to see whether genes involved in distal SNP–CpG pairs are closer in the FI network than expected by chance. Out of the 4,131 gene–gene pairs that can be formed by joining one SNP-annotated gene and one of its associated CpG-annotated genes, 1,140 pairs had both genes mapped to FI networks. The shortest path between these pairs of genes was calculated to be 3.48 (meaning that they are connected but only through another 2.48 genes on average), no different from what is expected from random pairs of connected genes (~3.50, estimated from 1,000 replicates, *P*=0.2 estimated from proportions). Only two pairs of genes display direct protein–protein interactions: UBE2N (annotated to rs6538421) and UBR4 (annotated to cg00223950); and ERBB4 (annotated to rs7599312) and SH3GLB2 (annotated to cg20548744).

### Expression quantitative loci associated with distal meQTLs

We considered the possibility that distal sites with differential CpG methylation could display differential allelic expression of transcripts that are in the vicinity of the CpG. We analysed independent genetic and transcriptomic data sets derived from 137 CD4 and 137 CD8 lymphocytes for the presence of eQTLs associated with the 1,387 unique SNPs involved in distal SNP–CpG methylations.

**Figure 2:**
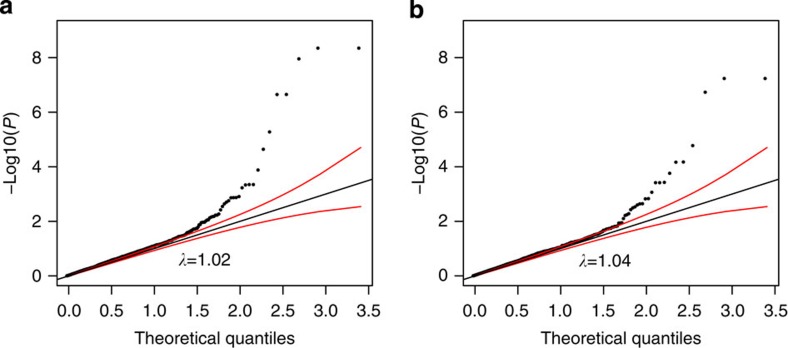
Quantile–quantile **plot of association levels of** ***trans*****-eQTL.** (**a**) *Trans*-eQTL in CD4; (**b**) *trans*-eQTL in CD8. Red lines are 95% confidence bands. *λ* is the inflation factor, the ratio of observed to expected medians.

Figure 2a,b displays quantile–quantile plots of significance levels against theoretical quantiles for associations between the SNPs and expression levels of distal protein-coding and non-coding transcripts found in their LD-defined neighbourhoods, in CD4 and CD8 cells. In each cell type, the weight of the distribution deviates towards greater levels of associations than predicted by chance, supporting the presence of enrichment of *trans*-eQTLs. Supplementary Data 2 lists the results of all tested *trans*-eQTL/gene pairs, and also present *cis* pairs for completeness.

In Battle *et al*.^24^, *trans*-eQTL (interchromosomal) were identified from a larger number of samples (922 DNA samples derived from whole blood) for 138 genes and distant eQTL (intrachromosomal eQTL, >1 Mb from the gene) for 269 genes, with 5 genes overlapping both lists. Six of these genes are also displaying significant meQTL with at least one annotated CpG site, either with the SNP itself or one in LD with it (smallest observed *R*^2^>0.70). These are: *PDE4DIP* (annotated to a distant eQTL), *DUSP22*, *PGLS*, *ZNF154*, *ZNF274* and *ZNF551* (annotated to *trans*-eQTLs).

### Relevance of meQTLs with respect to autoimmune diseases

We have compared the list of proximal and distal meQTLs with autoimmune disease (AID)-associated SNPs. Using Immunobase (https://www.immunobase.org), we generated a list of the currently known associated SNPs (non-major histocompatibility complex). In total, we found 552 associations across 11 AID (indexed in Immunobase; considering Crohn’s disease and ulcerative colitis together as inflammatory bowel diseases) involving 512 unique SNPs. Furthermore, since some of the associated SNPs in one disease are in high LD (*R*^2^>0.8) with associated SNPs in other diseases, this list covers a total of 447 associated loci (two are on the X chromosome). These 512 AID-associated SNPs overlap or are highly correlated (*R*^2^>0.8) with 200 of the proximal meQTLs (associated with a total of 561 CpG sites) and 14 of the distal meQTLs (associated with a total of 24 CpG sites) (Supplementary Data 4). Alternatively, 161/552 (29%) of the reported AID associations overlap or correlate with at least one proximal meQTL, and 9/552 (2%) overlap or correlate with at least one of the distal SNPs. In terms of the 447 independent loci, these percentages are, respectively, 115/447 (26%) and 8/447 (2%). This descriptive analysis illustrates that localization of disease-associated genes can benefit from the meQTL annotation of disease-associated SNPs.

### Case studies of three *trans*-meQTL loci

SENP7 (Sentrin/small ubiquitin-like modifier (SUMO)-specific protease 7) is an isopeptidase, which catalyses the deSUMOylation of SUMO2/3-conjugated proteins. The reversible post-translational SUMO modifications of proteins regulate many cellular processes including replication, transcription, recombination, chromosome segregation and cytokinesis. SENP7 interacts with BCL6, CBX5, KAP1 and HP1 alpha proteins, which are involved in epigenetic repression. SENP7 was recently described to promote chromatin relaxation in response to DNA damage, for repair and for cellular resistance to DNA-damaging agents^25^. Depletion of SENP7 results in spread of heterochromatin factors and condensed chromatin^25^.

**Figure 3:**
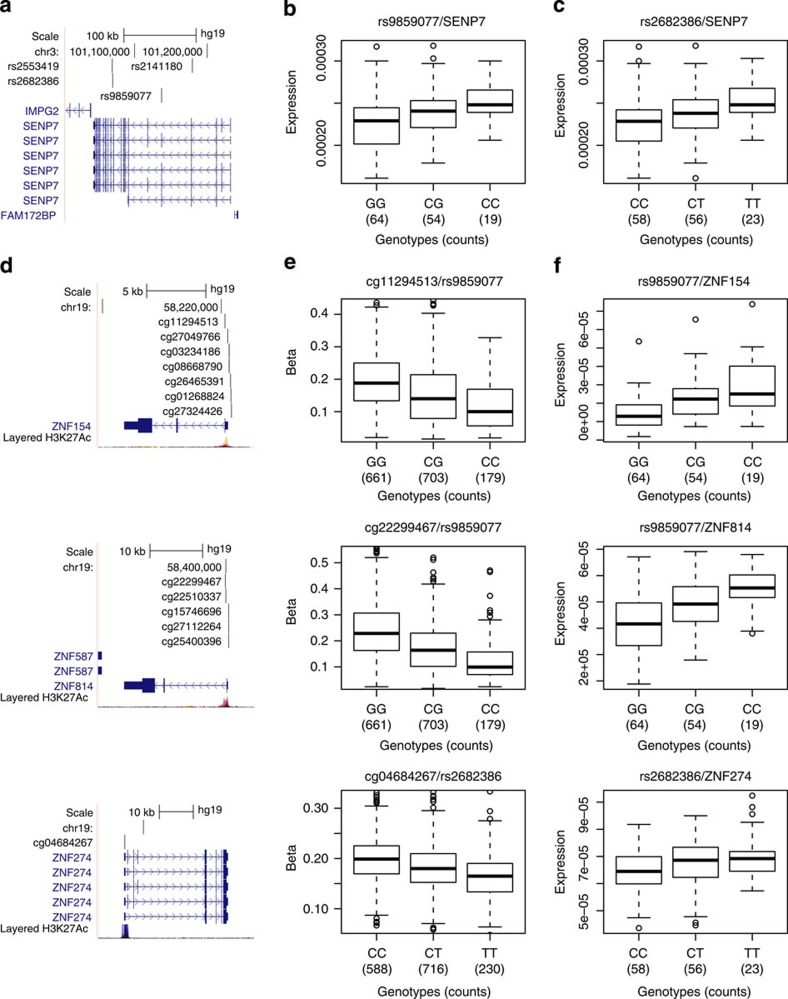
The *SENP7* locus and its distally associated CpG sites. (**a**) UCSC browser illustration of the gene structure and location of SNPs associated with distal CpG sites. (**b**,**c**) Association in *cis* between expression of *SENP7* in CD4 cells and (**b**) rs9859077, (**c**) rs2682386. (**d**) UCSC browser illustrations of selected associated distal CpG sites and their annotated genes (from top to bottom: *ZNF154*, *ZNF814* and *ZNF274*). (**e**) Corresponding boxplot representations of the SNP/CpG associations. (**f**) Corresponding boxplot representations of the association in *trans* between expression of the genes in CD4 cells with SNPs.

In our data set, four intronic SNPs (rs2553419, rs2682386, rs9859077 and rs2141180) of the *SENP7* gene in high LD with each other correlate with the methylation levels at numerous CpG sites (replicated in at least MARTHA or F5L for the large majority; Supplementary Data 2); these SNPs correlate with *cis*-acting regulation of *SENP7* expression in CD4 and CD8 lymphocytes (FDR<5%; *P*_Wald_<0.00015 derived from a Wald test; Supplementary Data 2) and *trans*-acting regulation of several distal genes, including *ZNF154*, *ZNF274* and *ZNF814* (Fig. 3; Supplementary Data 2), which reside within a ~250-kb region on chromosome 19, as well as *ZNF268* (chromosome 12) and *LDHD* (chromosome 16). Elevated levels of *SENP7* transcripts are associated with reduced CpG methylation located in 5′-flanking regions of *ZNF154*, *ZNF274* and *ZNF814* and increased transcript levels of these genes (*P*_Wald_<0.006); other genes whose CpG methylation is associated with *SENP7* intronic SNPs show similar trends of expression changes that do not meet the significance threshold (FDR>5%). These data indicate that SENP7 transcript levels regulate methylation and transcript expression of *trans*-meQTLs at multiple sites.

The identification of *SENP7*-regulated genes such as *ZNF154* potentially extends the understanding of SENP7 function and linkages to cancer processes. ZNF154, a putative TF expressed in many tissues, was initially identified as commonly deleted in thyroid adenoma^26^. Hypermethylation at the CpG island in the promoter region of *ZNF154*, and negative correlation between methylation and gene expression were recently described in serous ovarian cancers, and in endometrioid ovarian and endometrial cancers^27^. Methylation-mediated repression of *ZNF154* in ovarian cancer is associated with poor overall survival^28^. In clear cell renal cell carcinoma^29^ and in bladder cancer *ZNF154* is hypermethylated, and *ZNF154* methylation is identified as a biomarker of bladder cancer recurrence^30^,^31^.

**Figure 4:**
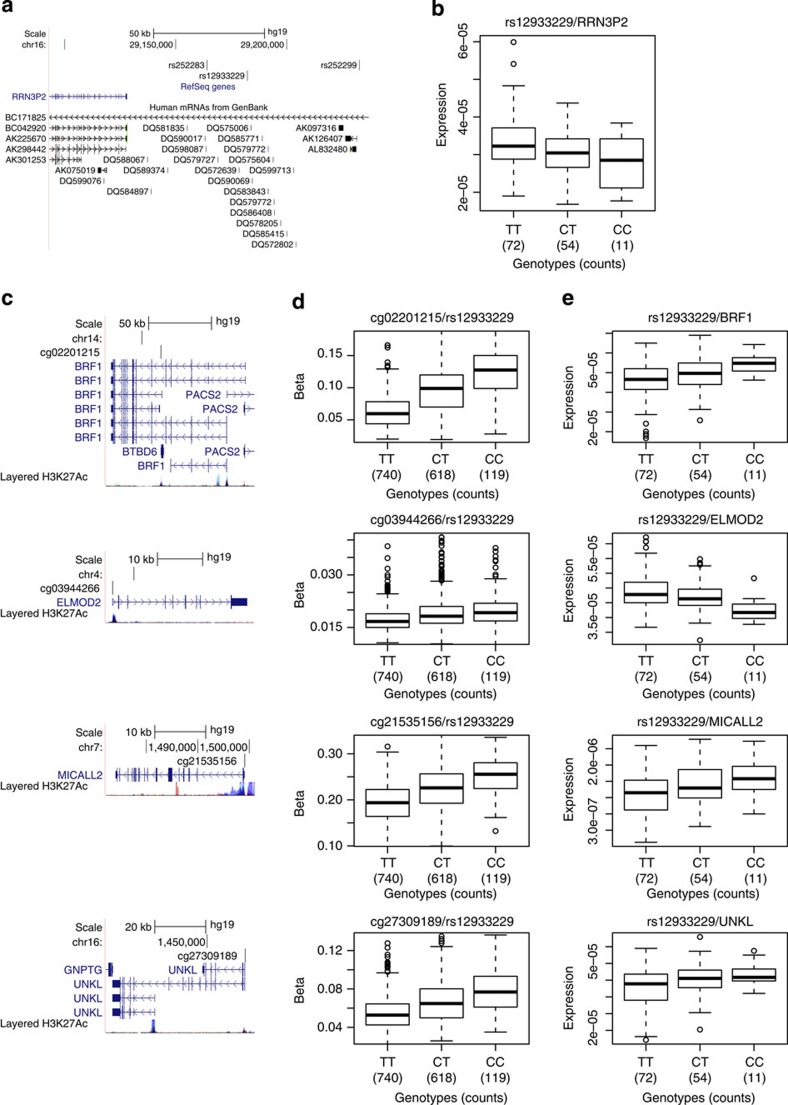
The *RRN3P2* locus and its distally associated CpG sites. (**a**) UCSC browser illustration of the gene structure and location of SNPs associated with distal CpG sites. (**b**) Association in *cis* between expression of *RRN3P2* in CD4 cells and rs12933229. (**c**) UCSC browser illustrations of selected associated distal CpG sites and their annotated genes (from top to bottom: *BRF1*, *ELMOD2*, *MICALL2* and *UNKL*). (**d**) Corresponding boxplot representations of the SNP/CpG associations. (**e**) Corresponding boxplot representations of the association in *trans* between expression of the genes in CD4 cells with rs12933229.

A chromosome 16 region of LD with rs12933229 (*R*^2^>0.5), which is devoid of protein-coding genes, is rich in piRNAs and contains *RRN3P2* (a long non-coding RNA gene), is a *trans*-meQTL locus associated with methylation at 20 distal CpG sites (all replicated in both MARTHA and F5L) residing on nine non-homologous chromosomes (Fig. 4 for selected genes whose expression are the most strongly associated with the SNP; Supplementary Data 5). Five of these *trans*-meQTL-associated regions have independently been reported^19^. Enrichment of the H3K27Ac histone marks and TF-binding sites as determined by Chip-seq assays^32^, as well as the presence of spliced expressed sequence tags (ESTs) indicate enhanced transcriptional activity in the region encompassing the piRNA cluster (Supplementary Fig. 3). piRNA clusters are usually transcribed as long single-stranded RNA and processed into mature piRNAs of 25–33 nucleotides in length^33^,^34^. The rs12933229 also correlates with *cis*-acting regulation of *RRN3P2* expression in CD4 and CD8 lymphocytes (*P*_Wald_=0.0012 and *P*_Wald_=0.038, respectively). Long non-coding RNA (linc) *cis*-eQTLs can also influence the expression levels of downstream genes^35^; in this case, *RRN3P2* could be a *cis*-regulator of piRNA transcription. Due to the described role of piRNA in methylation, we surmise that the *trans*-regulatory effects on methylation at distal genes may be mediated by one or more of the 21 piRNA genes (which were not measured in the lymphocyte RNA expression studies) in the ~56-kb region harbouring SNPs in LD with rs12933229. The Piwi/piRNA pathway has been described to promote heterochromatin formation, DNA methylation, transcriptional and post-transcriptional gene silencing and transcriptional activation by promoting euchromatic features^34^,^36^,^37^.

We observe that for 19/20 (95%) of distal CpG sites that are associated with rs12933229, the *β*-value increases with each copy of the C allele at rs12933229, suggesting that the *trans*-effect is consistent in how it distally regulates methylation (Supplementary Data 5). Interestingly, there are nine transcripts in the vicinity of these distal CpGs that show expression level changes in CD4 cells that are associated with rs12933229 (*P*_Wald_<5%) (Supplementary Data 5). Two genes (*ELMOD2* and *SLC35A3*) show increased expression correlating with hypomethylation of their respective CpGs (cg03944266 and cg16383001). However, we observe increased gene expression correlating with CpG hypermethylation for seven genes (*STARD3*/cg01325958, *BRF1*/cg02201215, *BTBD6*/cg02201215, *MAP3K14*/cg07370464, *GPC2*/cg13921324, *MICALL2*/cg21535156 and *UNKL*/cg27309189). This unusual association of increased CpG methylation and increased expression of local transcripts does not follow the classical ‘negative correlations’ between DNA methylation and gene expression. Recent studies comparing DNA methylation and gene expression have reported this paradox^5^,^14^,^18^,^38^, and surmised that the position of the CpG within the gene body may be relevant, with DNA methylation in promoters being negatively correlated, while DNA methylation within the gene body^39^ or 3′untranslated regions^40^ being positively correlated with gene expression, through mechanisms not yet explained. In our data set, we do not observe consistent patterns between the position of the CpG and the relationships between CpG methylation and gene expression. Further efforts are needed to tease out these effects resulting from a number of possible mechanisms involving chromatin state, histone modifications, TF availability and binding, as well as post-transcriptional regulation, including changes in RNA decay and stability by RNA-binding proteins and levels of small RNAs.

**Figure 5:**
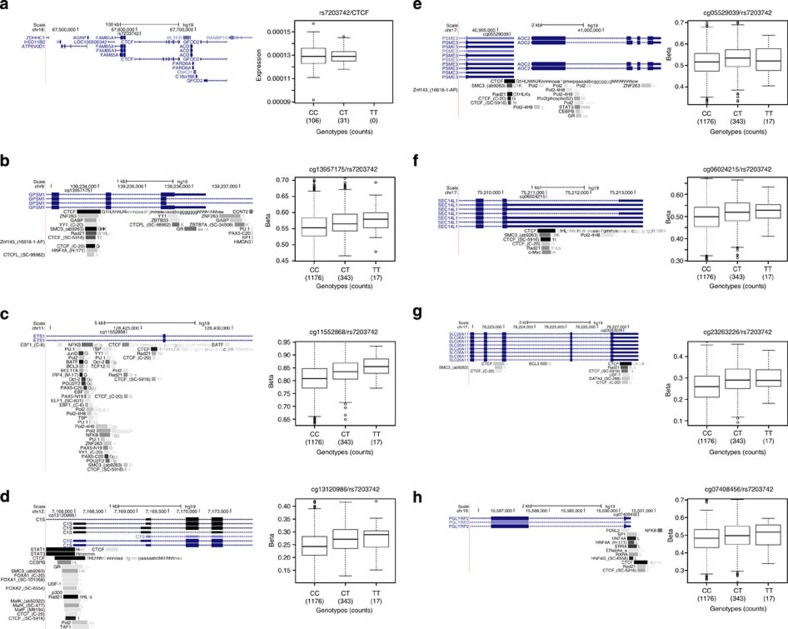
The *CTCF* locus and its distally associated CpG sites. (**a**) UCSC browser illustration of the gene structure and location of SNPs associated with distal CpG sites, and boxplot representation of the association in *cis* between CTCF expression and SNP genotypes. (**b**–**h**) Distally associated CpG sites, their gene annotations and boxplot representations of SNP–CpG associations. The Transcription Factor ChIP-seq from ENCODE tracks are displayed in the UCSC browser illustrations to indicate CTCF-binding sites.

CTCF (CCCTC-binding factor) is a zinc-finger DNA-binding protein that functions in transcription (activation and repression), RNA splicing, insulator activity, chromatin architecture and in genomic imprinting^41^. It is estimated that there are ~30,000 CTCF-binding sites in the human genome^42^. The rs7203742 SNP in the intron of *CTCF* is associated with methylation levels at 14 CpG sites across the genome (all replicated in MARTHA, the majority replicated in F5L; Supplementary Data 2). For *AOC2/PSME3*, *SEC14L1*, *PGLYRP2*, *ETS1*, *C1S*, *GPSM1* and *SLC26A11* genes (Fig. 5), and in regions without an annotated transcript, the rs7203742-associated CpG sites overlaps with CTCF-binding sites. This is consistent with observations that TFs can influence methylation at their binding sites^18^,^43^. These CpG sites reside in the promoter regions, introns and exons (untranslated and coding) of the associated genes. The CTCF binding to non-methylated/hypomethylated CpG sites, and its inhibition of binding to methylated DNA in concert with TFs and distal enhancers could result in repression or activation of the resident transcript. Such CTCF-mediated transcriptional repression and activation have been described for the imprinting control region of *Igf2/H19* genes^41^,^44^.

In both the CD4 and CD8 lymphocyte expression data sets described above, we did not detect significant eQTLs associations. However, there are published reports that independently validate CTCF-related meQTLs. Transcriptome sequencing on lymphocyte messenger RNA from patients with intellectual disability (MIM 604167), harbouring mutant CTCF, and healthy individuals identified *SEC14L1* as the top-ranked dysregulated gene in patients^45^. Conditional deletion of *Ctcf* in mice myeloid cells resulted in 4.77-fold upregulation of *C1s* gene in *Ctcf*-deficient macrophages^46^. These data substantiate the repression and activation effects of CTCF on the expression of *SEC14L1* and *C1s* genes, respectively.

## Discussion

In this study, we demonstrate that there is extensive long-range regulation of CpG methylation associated with genetic variation in the genome. A strength of the study is the large sample size used for the discovery of meQTL loci. The vast majority of our distal associations were replicated independently, using a variety of sampling designs (case–controls, case-only and family based), disease (colon cancer or venous thrombosis (VT)) normalization and statistical framework (linear models with fixed-only or mixed effects; using the methylation values as the dependent variable or their logit-tranformations; using LRTs or robust Wald tests) and cell type heterogeneity correction (principal component analysis; measured); they are statistically robust. While this data set is neither comprehensive due the incompleteness of the CpG sites that were ascertained and the single source of tissue that was used, the implications of the results are daunting, as it is quite likely that the >1,000 meQTLs represent the tip of the iceberg in regards to the numbers of distal pairs linking genetic variants and with CpG methylation that are present in the human genome. Revisiting the data sets presented here using more powerful strategies or combining them, as well as other data sets, by means of meta-analyses would likely reveal other meQTLs and their distal targets.

We were able to further analyse our meQTL results in expression data sets derived in an independent set of genotyped lymphocytes. This allowed us to identify many genetic variants that are associated with both differential CpG methylation and expression affecting distal genes. We note that future studies would benefit from having methylation and expression data sets generated in the same tissue panels, as it could enhance the detection of interrelated effects on transcription and expression. Nonetheless, the current data sets provided numerous examples of this, with *SENP7* being a prime example.

We focused our analyses on loci that correlate with CpG methylation at multiple distal sites. This revealed a number of possible regulators including SENP7, CTCF and a piRNA cluster, although the putative mechanism of *trans*-regulation appears to be different for each. While the current concepts on how each of the regulators may affect distal regulation is only partially known, the widespread functions that these genes are reported to have on gene transcription and chromatin architecture are consistent with our observations.

Finally, this study provides new avenues to understand molecular processes that are coordinated by genetic and epigenetic mechanisms, and study genetic loci associated with disease predisposition.

## Methods

### Methylation sample description

The cases and controls used in this study were enrolled in phase I of the OFCCR^6^,^47^. Briefly, probands were selected from incident colorectal cancer cases identified between 1 July 1997 and 30 June 2000 from the population-based Ontario Cancer Registry. Controls were recruited through telephone interviews in randomly selected households in Ontario. A subset of OFCCR cases and controls were used in the Assessment of Risk for Colorectal Cancer Tumours in Canada study that led to the identification of a genetic risk factor for colon cancer in the 8q24 region^7^ and are used in this study. Informed consent was obtained from all participants.

### Methylation profiling

We profiled 2,203 samples from 2,101 unique donors (1,103 cases of colorectal cancer and 998 controls). Lymphocyte pellets were extracted from whole blood using Ficoll-Paque PLUS (GE Healthcare). DNA was extracted from lymphocytes using phenol–chloroform or the Qiagen Mini-Amp DNA kit, except for 99 samples (90 cases, 9 controls), for which DNA was extracted from lymphoblastoid cell lines. We used 15 μl of DNA from all samples at concentrations averaging 90 ng μl^−1^ (20 ng μl^−1^ s.e.). DNA samples were bisulfite-converted using the EZ-96 DNA Methylation-Gold Kit (Zymo Research, Orange, CA); 4 μl of bisulfite-treated DNA was then analysed on the HumanMethylation450 BeadChip from Illumina according to the manufacturer’s protocol. Except for the case/control status, the plating of the DNA samples was not based on any specific characteristics that they might share (for example, gender and age), effectively randomizing these factors on the arrays. Most cases and most controls were plated and processed separately, except for 125 cases that were matched to 125 controls on 125 rows of 36 arrays; for these samples, batch and position effects are controlled for by design.

### Calculation of methylation ratios from intensities

We calculated the methylation ratios (proportion of molecules that are methylated at a given site, also known as *β*-value) as *β*=min(*M*,1)/(min(*M*,1)+min(*U*,1)) where *M* and *U* are, respectively, the methylated and unmethylated intensity signals (possibly background-corrected signals). This is a slight modification of GenomeStudio’s calculation: Illumina defines the ratio as *β*=min(*M*,0)/(min(*M*,0)+min(*U*,0)+100). In this expression, the offset (100) artificially moves the methylation value away from 1. Moreover, the possibility of the numerator being 0 makes the resulting distribution of *β*-values both discrete and continuous (with point mass at 0). We prefer our expression for *β*, which results in a continuous distribution that does not bias against a fully methylated state.

### Processing of idat files and normalization

We used functions from the methylumi package from Bioconductor to read the idat files and write intensity values to text files. We compared the methylation values (the proportion of molecules that are methylated at a given site, also known as *β*-value) of a number of normalization strategies by focusing on 64 samples that were done in duplicate or triplicate (129 possible pairs after sample exclusions) (see Supplementary Methods and Supplementary Figs 4–11). On the basis of these comparisons, we hereafter use methylation values derived from data that was background-corrected using NOOB^48^ followed by colour adjustments using Illumina’s normalization probes and algorithms; BMIQ^49^ was then applied to the set of *β*-values.

### Principal component analysis

We performed a principal component analysis by first calculating the covariance matrix between all samples using only the most variable autosomal CpG sites, measured in terms of their 95% reference range: the range of methylation values observed in the central 95% of the samples, or more precisely the difference between the 97.5 and 2.5% percentiles. Using a 95% reference range of at least 0.20, 131,045 CpG sites were used in the covariance matrix calculation. Together, the top three principal components explain over 92.5% of the total variance. Each subsequent vector does not add substantially to the variance explained: 98 vectors would be necessary to explain 95% of the total variance.

### Sample exclusion

We excluded from association analyses: (1) samples for which DNA was extracted from lymphoblastoid cell lines (99 samples including 1 in duplicate); (2) samples that were outliers with respect to any one of the internal control probes (excluding probes designed to evaluate the background noise and probes designed to normalize the data), where an outlier is defined as any value more than three times the interquartile range away from the closest quartile (124 samples); (3) samples that were outliers with respect to any one of the first 12 principal components (corresponding to the approximate location of the elbow of the eigenvalue scree plot), recomputed after exclusion of the samples in steps 1 and 2 (26 samples; see Supplementary Methods); (4) samples that were not of non-Hispanic white ancestry, either self-declared or by investigation of genetic ancestry using genome-wide SNP data (164 samples); and (5) samples with intensities on the X or Y chromosome inconsistent with their gender (seven samples). These overlapping counts sum up to 389 unique samples that were excluded. For duplicate samples, we only kept the one with the smallest number of sites with a detection *P* value (see below) less than 0.01. After exclusion of samples, we were left with 1,748 samples: 898 cases (females: 58.9%; mean age: 63±8.1 s.d.) and 850 controls (females: 42%; mean age: 64.3±8.2 s.d.).

### Probe exclusion

The detection *P* value is a quantitative assessment of the probability that the total intensity, for a given sample at a given site, can be distinguished from background noise, and is included along with the intensity values. All *β*-values with a detection *P* value less than 1% were treated as missing data. Sites with more than 1% missing values after sample exclusion were discarded (13,032 sites).

### Polymorphic sites

We excluded from SNP–CpG-methylation association analyses all CpG sites that were polymorphic at the cytosine or at the guanine base, and, in the case of Infinium I probes, had a SNP at the position where single-base extension occurs (the base before the CpG cytosine), irrespective of the allele frequency. We moreover excluded CpG sites for which a SNP resided elsewhere within the probe target sequence as long as its MAF was above 5%. Allele frequency estimates were extracted from the samples of European ancestry (EUR; 379 samples) of the 1000 Genomes Project^50^.

### Probe cross-reactivity

We excluded CpG sites from SNP–CpG-methylation association analyses if their probe sequences aligned to multiple positions with ≥90% identity; misalignments were ignored if the terminal nucleotide of the probe was a mismatch (preventing single-base extension), if the probe aligned with gaps or if the probe mapped onto an alternative assembly of the target chromosome. See ref. 8 for additional details.

### Cell-type proportions

Because differences in cell-type proportions between DNA samples can confound association results^51^, we adjusted our analyses using a surrogate for cell-type proportions derived from 49 differentially methylated CpG sites present on the HumanMehtylation450 array that have the ability of discriminating between blood cell types^52^. As a surrogate for cell-type proportions, and to reduce the number of variables, we used the first two principal components associated with these 49 sites that together explain over 90% of the total variance.

To verify that the first two principal components that we derived from the list of 49 differentially methylated CpG sites^52^ can indeed serve as a surrogate for blood cell proportions, we tested for associations between the principal components and the methylation levels at all of our sites, adjusting our analyses for sex, age, arrays and position of the samples on the arrays. We selected the top 10% of the sites that showed the strongest associations (46,000 sites, all associated at levels *P*<10^−100^) and extracted these sites in data sets of purified human leukocyte subtypes^53^ (GEO accession: GSE39981); 2,605 sites were overlapping. A dendrogram representation of our top sites in this data set^53^ reveals clear clustering of samples according to cell types, indicating a good ability for principal components to discriminate between samples with different cell compositions (Supplementary Fig. 1).

### Genetic variants and inter-individual levels of methylation

For each CpG site, we looked for SNPs that were associated with inter-individual methylation levels; we treated the methylation levels as a quantitative trait, and searched the genome for quantitative trait loci (meQTL) that explained a substantial proportion of the methylation variance at that site. We used SNP data from a colon cancer genome-wide association study^7^ (a 1536 GoldenGate panel from Illumina; the 10-k coding-SNP array from Affymetrix/ParAllele; the Human Mapping 100-k set from Affymetrix) all combined and complemented with the Human Mapping 500-k set from Affymetrix. When combining SNPs genotyped on multiple platforms or arrays, SNPs with more than 0.5% discordant calls were discarded; otherwise discordant calls were considered to be missing calls. SNPs with minor allele frequencies less than 5% in either the cases or the controls were ignored, as well as SNPs with genotypic frequencies inconsistent with Hardy–Weinberg equilibrium at *P*<10^−4^ in all samples and SNPs with call rates less than 90%; in all, 410,203 autosomal SNPs were available for pairwise comparison with 380,189 autosomal methylation sites.

We classify a SNP–CpG association as proximal if the SNP and the CpG site are separated by no more than 1 Mbp on the same homologous chromosome and as distal otherwise. Over 103 million SNP–CpG pairs were proximal candidates, while over 155 billion pairs were distal candidates. Preliminary analyses in cases and controls separately revealed over 80% overlap in significant results at stringent significance thresholds; we thus combined cases and controls to increase power.

We analysed the SNP–CpG pairs using a linear regression framework implemented in R: the *β*-value of a methylation site was taken as the independent variable, genotypes (number of minor alleles) at a SNP were taken as the values of the independent variable and the model included sex, age, case/control status, cell-type proportion surrogate, batch (plate) and position of the sample on its array as covariates. Significance of the SNP was calculated from a LRT (*P*_LRT_). Rare homozygous genotypes (count of less than 10) were combined with heterozygotes. Following this analysis, we report SNP–CpG associations consistent with an FDR (ref. 9) <5%, and report the effect size as the proportion *R*^2^ of the CpG methylation variance that is explained by the SNP, among the variance not already explained by the covariates.

We stratify results based on the inter-individual variability of the CpG sites. As a measure of variability, we used the 95%-reference range (the difference between the most and least methylated individuals, among 95% of the individual forming the central distribution of methylation values), which is less sensitive to outliers than the full range and more readily interpretable than the s.d.

All genomic positions are reported with respect to the hg19/GRCh37 coordinates.

A circular representation of distal SNP–CpG associations with ideograms was plotted using CIRCOS^54^.

### SNP–CpG replication sets

We sought to replicate distal SNP–CpG associations using two sets. (1) F5L: a set of 227 French–Canadian individuals belonging to five extended families (each ascertained through a single proband with idiopathic VT and carrying the factor V Leiden mutation^55^. These whole-blood samples were profiled on the HumanMethylation450 array and genotyped on the Human660W-Quad array from Illumina. We chose proxy SNPs from the Human660W-Quad array that are in high LD with SNPs involved in distal SNP–CpG associations when the latter were not present on the Illumina array; to seek consistency in the direction of association, we report phasing information and strength of LD between the pairs of SNPs (based on 1000 Genomes EUR data). (2) MARTHA: a set of 350 unrelated cases of VT patients of European ancestry (primarily of French descent) from the MARseille THrombosis Association study^55^,^56^. These whole-blood samples were profiled on the HumanMethylation450 array and genotyped on the Human610-Quad array from Illumina. Imputation of ungenotyped SNPs was performed using MACH v1.0.16a and minimac v4.4.3 (ref. 57) with their accompanying 1000 Genomes-based reference set^58^. We report the *R*^2^ measure of imputation accuracy.

All three sets (OFCCR, F5L and MARTHA) were analysed independently by the different groups providing the data, using methods and software seen as most appropriate for their own samples. Both F5L and MARTHA methylation data were normalized similarly to the OFCCR data set, with the exception that SWAN^59^ was used instead of BMIQ. For F5L, methylation values of the different CpG sites (beta values) were first logit-transformed and modelled as a linear function of the number of SNP alleles in a linear mixed regression model that included a random effect to account for the relatedness of the samples (implemented in GEMMA^60^); the model was furthermore adjusted for age, sex and cell-type proportions^61^. Significance was assessed using a LRT. For MARTHA, a linear regression model was used where the methylation values were taken as the outcome that was modelled using the allele dosage (from imputation) as the predictor, while adjusted for age, sex, batch, array, position of the sample on its array and measured cell-type compositions (lymphocytes, monocytes, polynuclear, eosinophils and basophils directly determined by ADVIA 120 Hematology System (Siemens Healthcare Diagnostics, Deerfield, IL)). Significance was assessed using a Wald test.

### SNP and CpG site annotations

We annotated sets of genes or features (for example, non-coding RNAs) to SNPs by selecting the smallest genomic region that contained all SNPs in high LD with a SNP of interest (at *R*^2^>0.5 according to 1000 Genomes^50^ data or HapMap release 22 when the SNP is not indexed in 1000 Genomes Project) and annotated to that SNP all genes (that were extended by 5 kb in their promoter regions) or features falling at least partially in that window. We counted the number of features falling in regions flanking our meQTLs. By randomly selecting a similar number of SNPs among the ones that entered the analysis and generating LD-defined regions, we calculated an average absolute fold enrichment of genomic features (fold enrichment of features per Mb) based on 1,000 replicates. Significance of feature fold-enrichment was calculated as the proportion of random replicates (sets of random regions) having a number of features greater or equal to the observed number of features in our meQTL regions. These fold enrichments are not meant to be compared with what is expected in random regions of the genome *per se*, but rather random regions of the subset of the genome that is covered by our set of SNPs, as well as SNPs in LD with them.

A CpG site was annotated to a gene if its location falls from within 1,500 bases from the gene’s transcription start sites up to and including its 3′untranslated region. This set of annotations is available with the HumanMethylation450 array documentation.

### Functional interaction networks

We downloaded version 2013 of the FI network from Reactome (www.reactome.org) that we analysed using functions from the igraph package of R (www.r-project.org).

### Expression data sets in CD4 and CD8 lymphocytes

The expression data from purified T-cell subpopulations was derived from multiple sclerosis (MS) patients (*n*=68) and healthy controls (*n*=67). MS cases were recruited through the Cambridge MS clinic and controls from the Cambridge (UK) BioResource (http://www.cambridgebioresource.org.uk). The study had approval from appropriate ethics committees and all subjects gave written informed consent. In total 50–80 ml of heparinized whole blood was collected from each individual and peripheral blood mononuclear cells (PBMCs) isolated using Ficoll density-gradient centrifugation. PBMCs were washed twice following Ficoll separation to remove platelet contamination. DNA was prepared from aliquots of the PBMCs using standard isolation protocols. CD4+ and CD8+ T cells were isolated using magnetic-activated cell sorting according to the manufacturers’ instructions (Miltenyi Biotec). Specifically CD3+ cells were negatively selected using a Pan T Cell Isolation Kit followed by positive selection of CD8+ cells with the remaining fraction representing CD4+ cells. The purity of the separated cells was checked by flow cytometry for a subset of the samples.

The isolated cells were immediately lysed in TRIzol reagent (Life Technologies) and stored at −80 °C prior to extraction. Total RNA was extracted according to the standard TRIzol protocol, and DNA contamination removed using DNase I treatment (Thermo Scientific). The extracted RNA was cleaned using the RNeasy MinElute Cleanup Kit (Qiagen), and the RNA integrity assessed using an Agilent 2100 Bioanalyzer and quantified using a Nanodrop 1000. Libraries for stranded total RNA-sequencing were prepared using the Illumina Stranded Total RNA protocol (RS-122-2301). Libraries were assessed using the Agilent 2100 Bioanalyzer.

Samples were indexed and sequenced on Illumina HiSeq 2000 (paired-end 2 × 100 bp). Raw reads were trimmed for quality (phred33≥30), length (*n*≥32) and Illumina adapters using Trimmomatic v. 0.22 (ref. 62). Filtered reads were aligned to the hg19 human reference (for human samples) using Tophat v. 1.4.1 (ref. 63) and bowtie v. 0.12.8 (ref. 64). Duplicates were marked in the bam file using Picard’s MarkDuplicates.jar v. 1.70 (http://picard.sourceforge.net). Raw read counts for ucsc and ensembl genes were obtained using htseq-count v. 0.5.3p9 (http://www-huber.embl.de/users/anders/HTSeq). FPKM values (fragments per kilobase of transcript per million fragments mapped) for ucsc and ensembl genes and transcripts were obtained using cufflinks v. 2.1.1 (ref. 65). To reduce the distorting effects of high data values in sequence data, we transformed all FPKM values with the inverse hyperbolic sine function asinh *x*=ln(*x*+√(*x*2+1)) and finally performed quantile normalization across signal-transformed FPKMs. Genotyping on the 137 samples was performed on Illumina Human 2.5 M arrays, and additional variants were imputed (Impute2) using 1000 Genomes (phase 1 integrated version 3) UK sample as reference. Association testing for genotype versus normalized FPKMs was carried out using PLINK^66^ using a linear framework for successfully imputed sites (info>0.8) without covariates, combining the cases and controls but separating CD4+ and CD8+ to independent data sets. Significance was calculated using a Wald statistic (*P*_Wald_).

## Author contributions

B.W.Z., S.G. and T.J.H. designed the epigenetic study. M.L. and T.J.H. conceived the MeQTL study. M.B., T.P. and S.S. designed the expression study. P.-E.M., F.G. and P.S.W. contributed data. M.L., B.G., M.G., D.A. and I.K. performed statistical analysis under the supervision of T.J.H., T.P., D.-A.T. and F.G. S.H.E.Z., M.W. and T.J.H. performed case studies of specific loci. M.L., S.H.E.Z., T.P. and T.J.H. wrote the article. All authors read and approved the final manuscript.

## Additional information

**Accession codes.** The OFCCR data were deposited in dbGaP under the accession number phs000779.v1.p1.

**How to cite this article:** Lemire, M. *et al*. Long-range epigenetic regulation is conferred by genetic variation located at thousands of independent loci. *Nat. Commun.* 6:6326 doi: 10.1038/ncomms7326 (2015).

## Supplementary Material

Supplementary Information Supplementary Figures 1-11, Supplementary Methods and Supplementary References

Supplementary Dataset 1 List of significant proximal SNP-CpG associations. The table shows proximal SNP-CpG pairs significant at FDR<5%, their positions, the proportion of methylation variance explained by the SNP, the mean methylation values stratified by genotype and genome annotations.

Supplementary Dataset 2 List of significant distal (trans) SNP-CpG associations, cis- and transeQTLs. Tab 1 shows distal SNP-CpG pairs significant at FDR<5%, their positions, the the proportion of methylation variance explained by the SNP, the mean methylation values stratified by genotype and genome annotations; Tab 2 shows association between SNPs and expression of genes in cis in CD4 and CD8 cells; Tab 3 shows association between SNP and expression of genes in trans in CD4 and CD8 cells.

Supplementary Dataset 3 List of 190 genes involved in epigenetic processes. Counts refer to the number of genes in the LD-defined neighborhood of SNPs involved in distal SNP-CpG associations

Supplementary Dataset 4 SNPs involved in autoimmune diseases. Counts refer to the number of SNPs involved in proximal SNP-CpG associations and distal (trans) SNP-CpG associations.

Supplementary Dataset 5 Distal associations between rs12933229 and methylation at 21 CpG sites. The table includes mean methylation values stratified by genotype and association with expression of annotated genes in CD4 and CD8 cells.

## Figures and Tables

**Table 1:** Number of CpG sites significantly associated with at least one proximal SNP.

***R***^2^	**All sites**	**Q1 (0–2.1%)**	**Q2 (2.1–4.7%)**	**Q3 (4.7–10.9%)**	**Q4 (10.9–21.4%)**	**Q5 (21.4–100%)**
2–5%	19,216	769	1,775	3,521	5,920	7,229
5–10%	14,494	355	951	2,234	4,508	6,444
10–25%	12,284	164	515	1,498	3,490	6,616
25–50%	4,960	22	89	370	1,133	3,345
50–75%	1,416	2	5	30	225	1,154
75%+	338	0	0	1	8	329
Total	52,708	1,312	3,335	7,654	15,284	25,117

SNP, single-nucleotide polymorphism.

Counts are stratified based on the site’s inter-individual variability and *R*^2^-value (proportion of methylation variance explained) between the SNP and CpG pairs. The variability of a site is measured as its 95%-reference range (the difference between the most and least methylated individuals, among 95% of the individuals forming the central distribution of methylation values) stratified in quintiles (Q1–Q5); percentages in brackets indicate the corresponding 95%-reference range values.

**Table 2:** Number of CpG sites significantly associated with at least one distal SNP.

***R***^2^	**All sites**	**Q1 (0–4.9%)**	**Q2 (4.9–7.0%)**	**Q3 (7.0–10.8%)**	**Q4 (10.8–17.8%)**	**Q5 (17.8–100%)**
2–5%	567	80	104	106	123	154
5–10%	676	68	122	154	149	183
10–25%	441	29	66	111	116	119
25–50%	177	6	38	38	45	50
50–75%	45	0	2	6	13	24
75%+	13	0	0	1	3	9
Total	1,919	183	332	416	449	539

SNP, single-nucleotide polymorphism.

Counts are stratified based on the site’s inter-individual variability and *R*^2^-value (proportion of methylation variance explained) between the SNP and CpG pairs. The variability of a site is measured as its 95%-reference range (the difference between the most and least methylated individuals, among 95% of the individuals forming the central distribution of methylation values) stratified in quintiles (Q1–Q5); percentages in brackets indicate the corresponding 95%-reference range values.
