# Supplementary material for: Long-range epigenetic regulation is conferred by genetic variation located at thousands of independent loci
Source: Nat Commun. 2015 Feb 26;6:6326. doi: 10.1038/ncomms7326 (PMC4351585; doi:10.1038/ncomms7326)
Supplement: Supplementary Information — Supplementary Figures 1-11, Supplementary Methods and Supplementary References [file ncomms7326-s1.pdf]

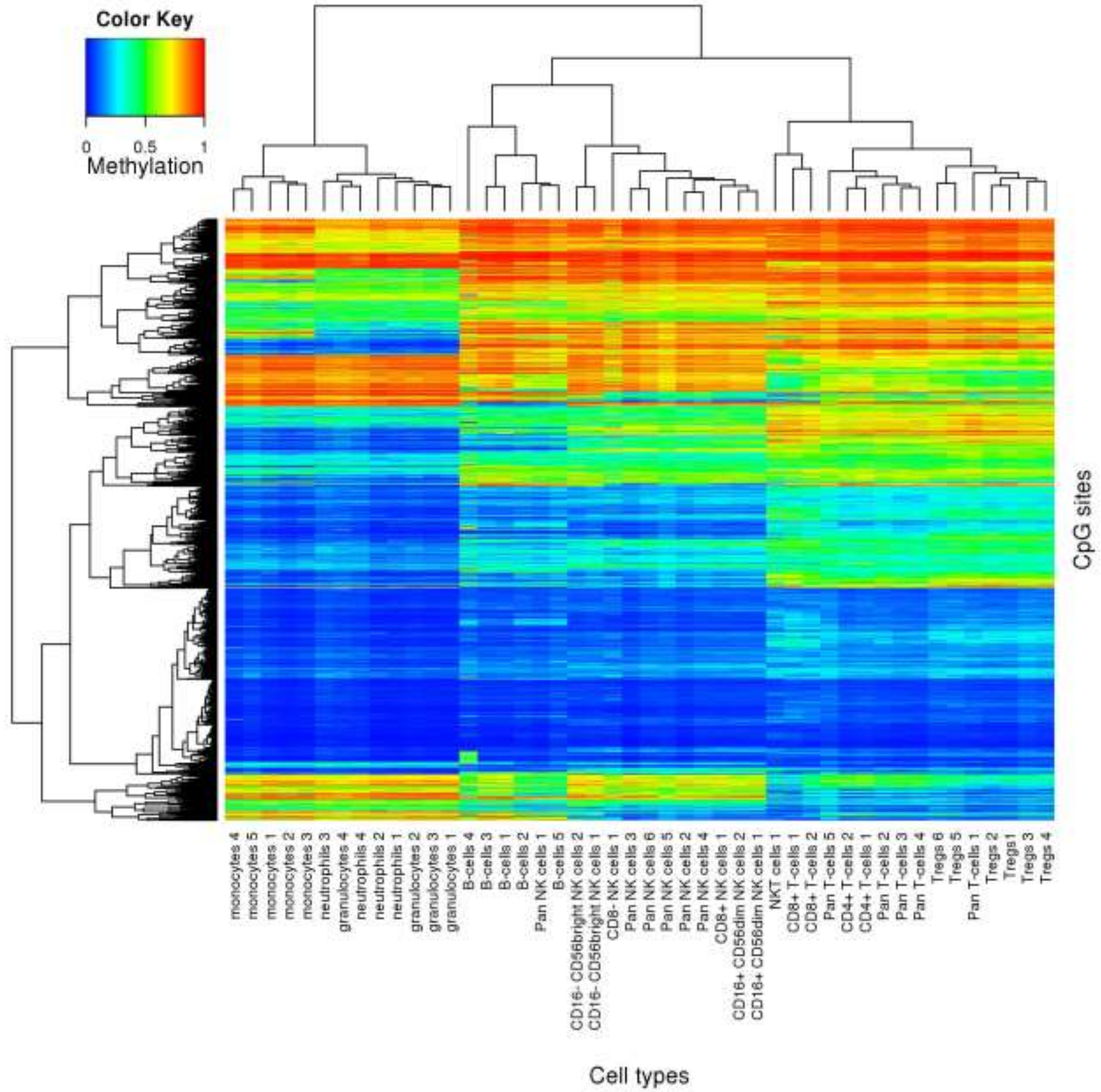

Supplementary Figure 1: Top CpG sites associated with blood cell type surrogates (principal components), evaluated in purified human leukocyte subtype methylation data sets.

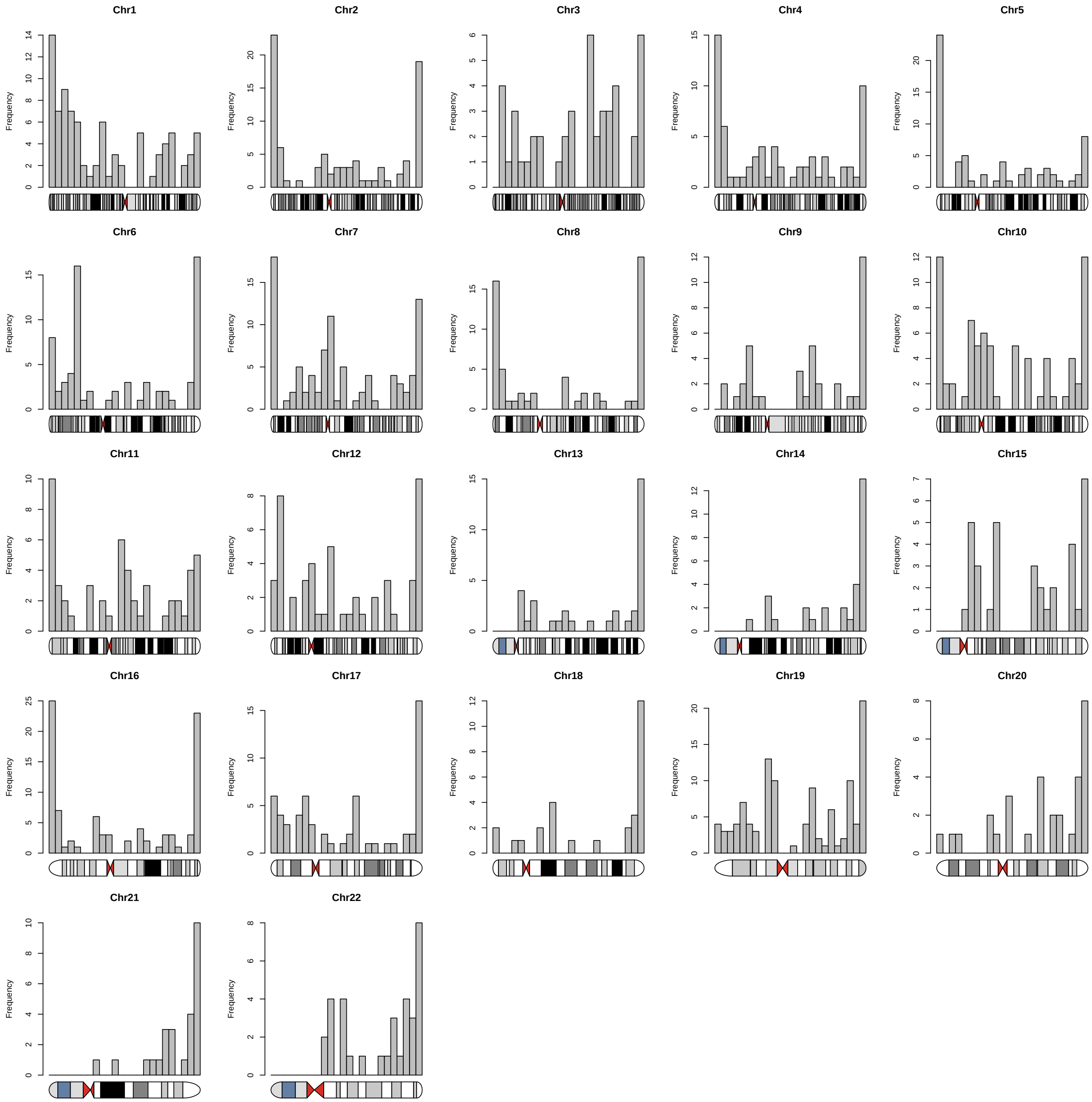

Supplementary Figure 2: Genomic positions of 1,388 SNPs involved in distal SNP-CpG associations, pruned for linkage disequilibrium.

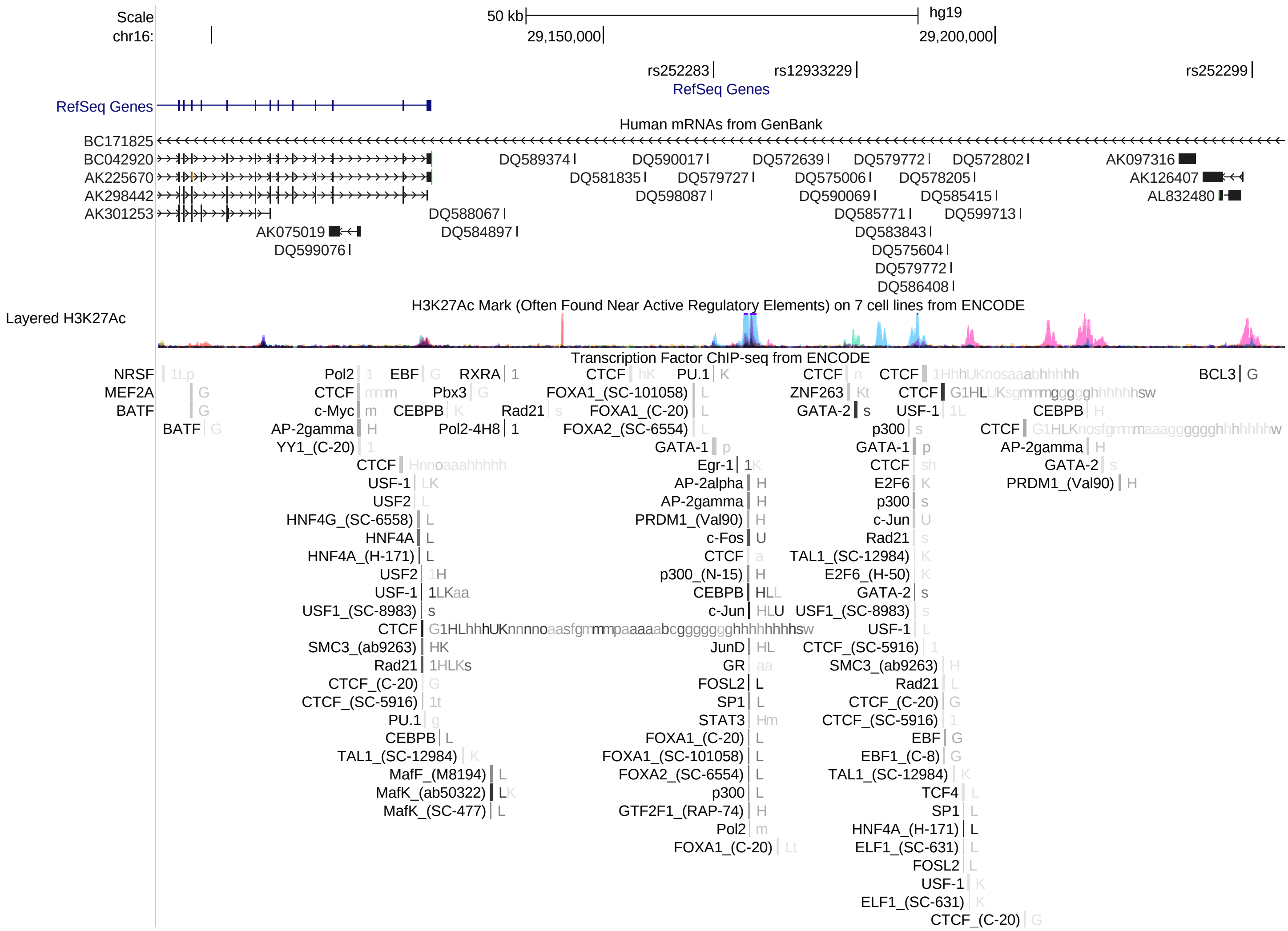

Supplementary Figure 3: UCSC browser illustration of the chr16:29093157-29236964 region.

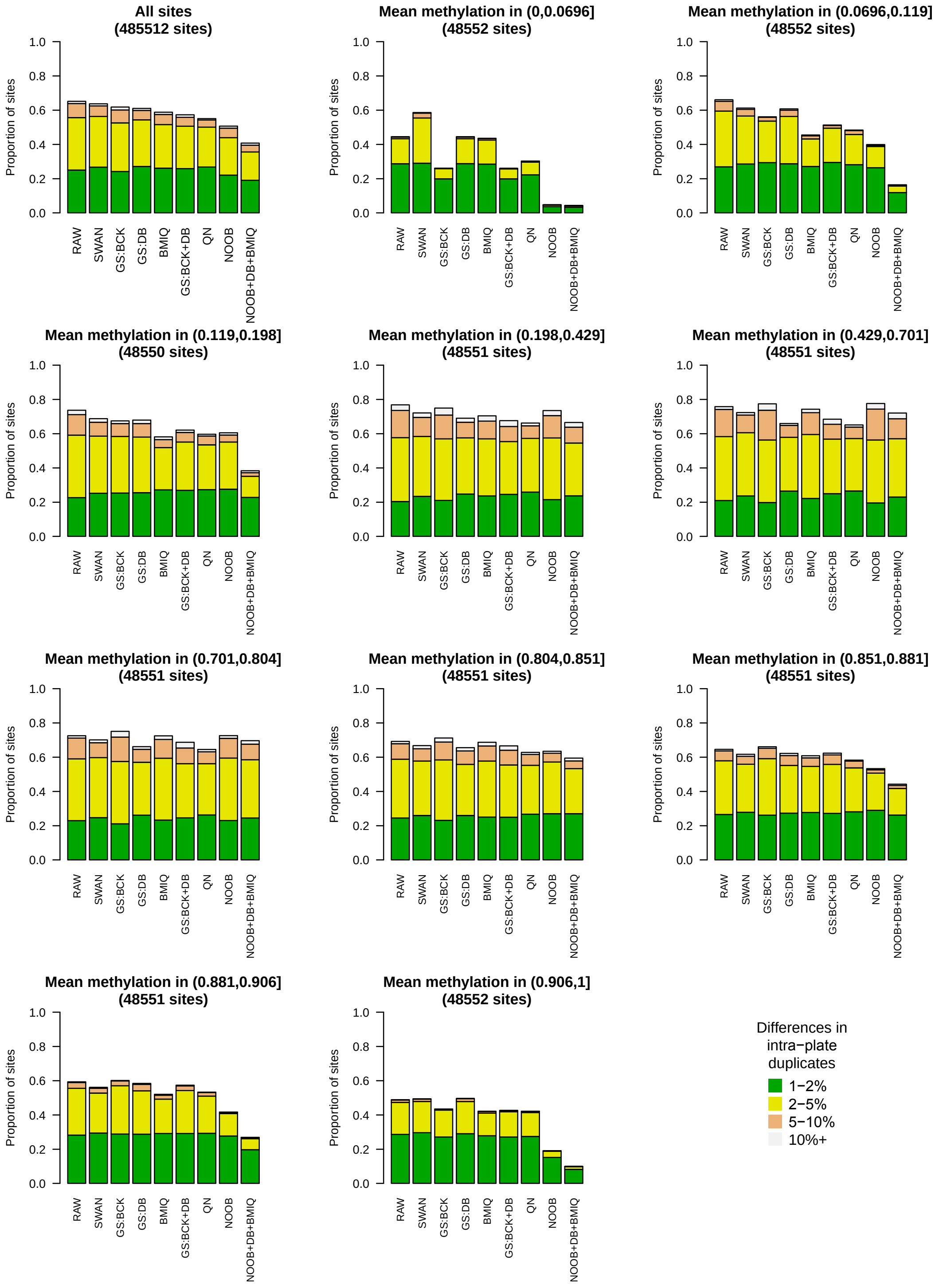

Supplementary Figure 4: Average proportion of all CpG sites having a β value that differs by 0.01-0.02 (green), 0.02-0.05 (yellow), 0.05-0.10 (pink) and more than 0.10 (light gray) in intra-plate duplicates, based on data transformation method. CpG sites are further stratified in deciles, based on the average methylation level of the site calculated from the raw data.

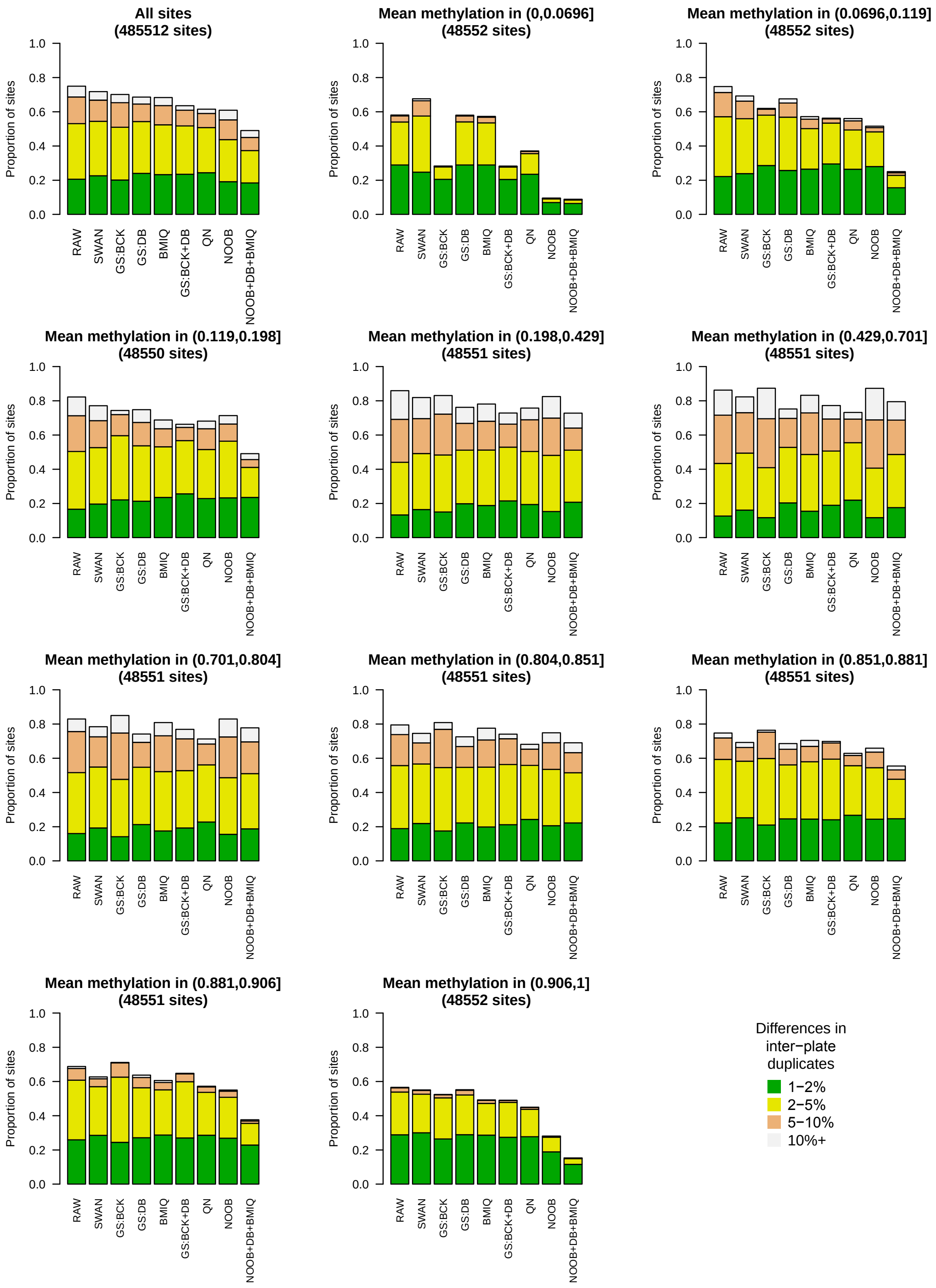

Supplementary Figure 5: Average proportion of all CpG sites having a β value that differs by 0.01-0.02 (green), 0.02-0.05 (yellow), 0.05-0.10 (pink) and more than 0.10 (light gray) in inter-plate duplicates, based on data transformation method. CpG sites are further stratified in deciles, based on the average methylation level of the site calculated from the raw data.

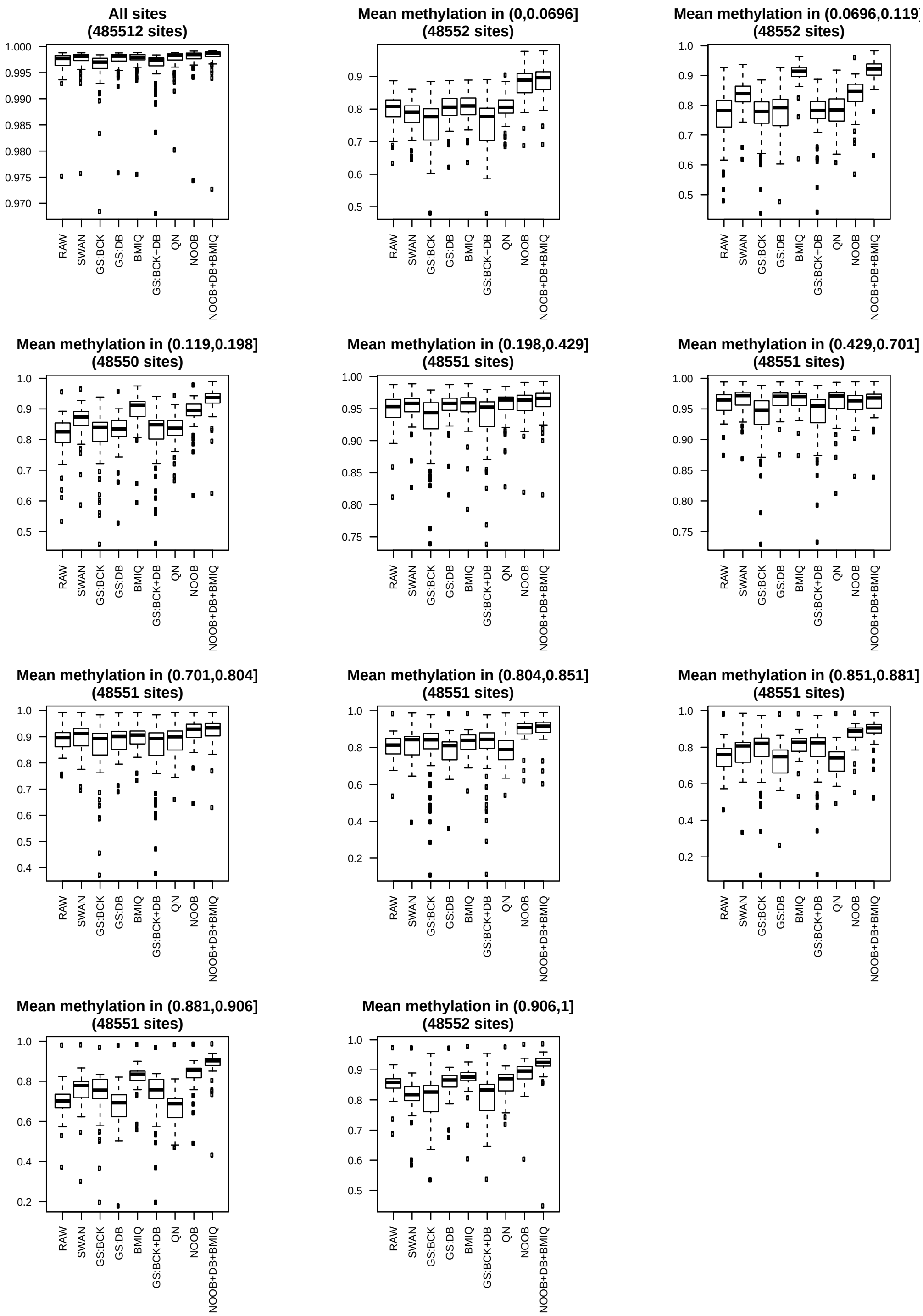

Supplementary Figure 6: Correlation between intra-plate duplicates based on data transformation method. CpG sites are further stratified in deciles, based on the average methylation level of the site calculated from the raw data.

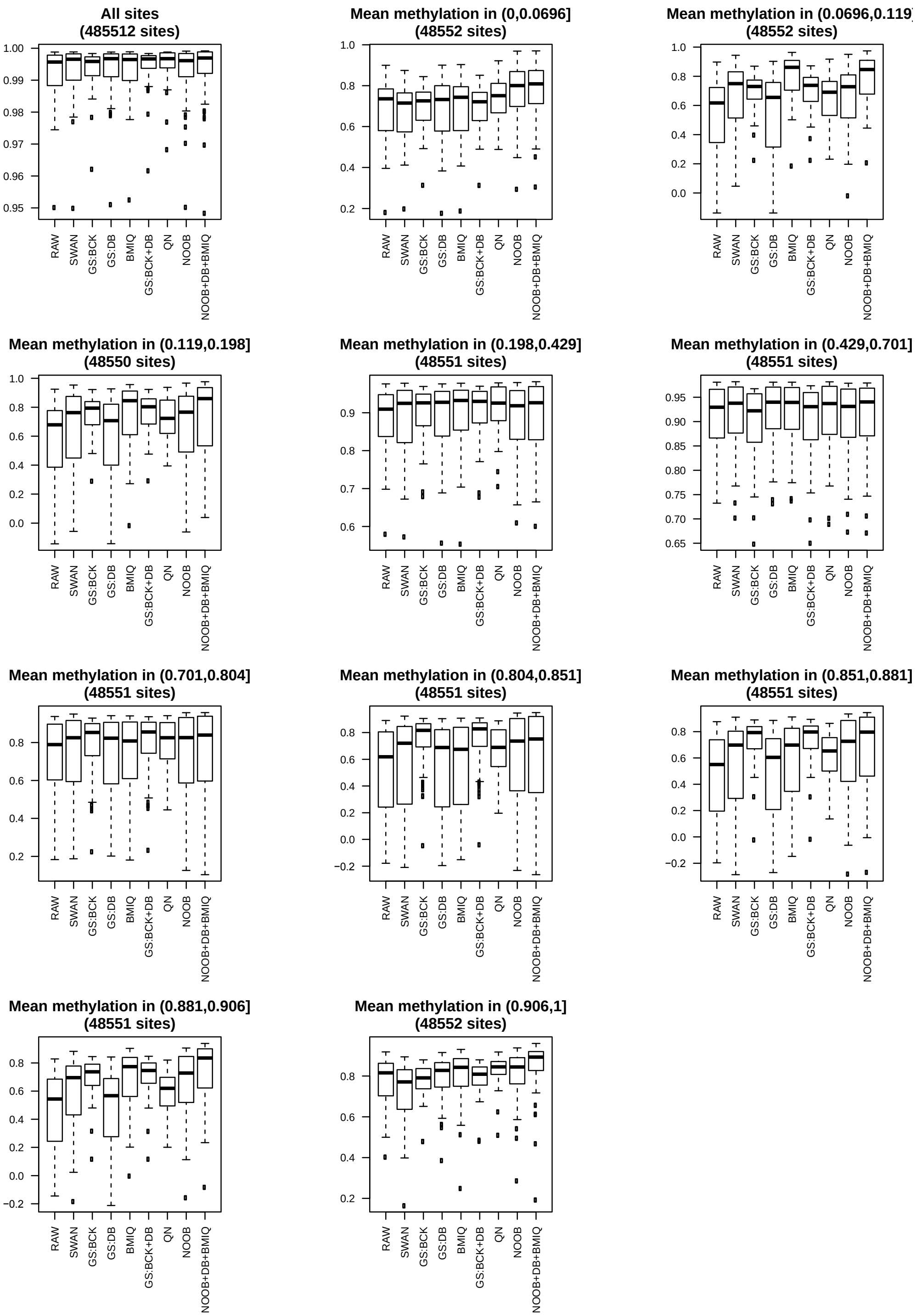

Supplementary Figure 7: Correlation between inter-plate duplicates based on data transformation method. CpG sites are further stratified in deciles, based on the average methylation level of the site calculated from the raw data.

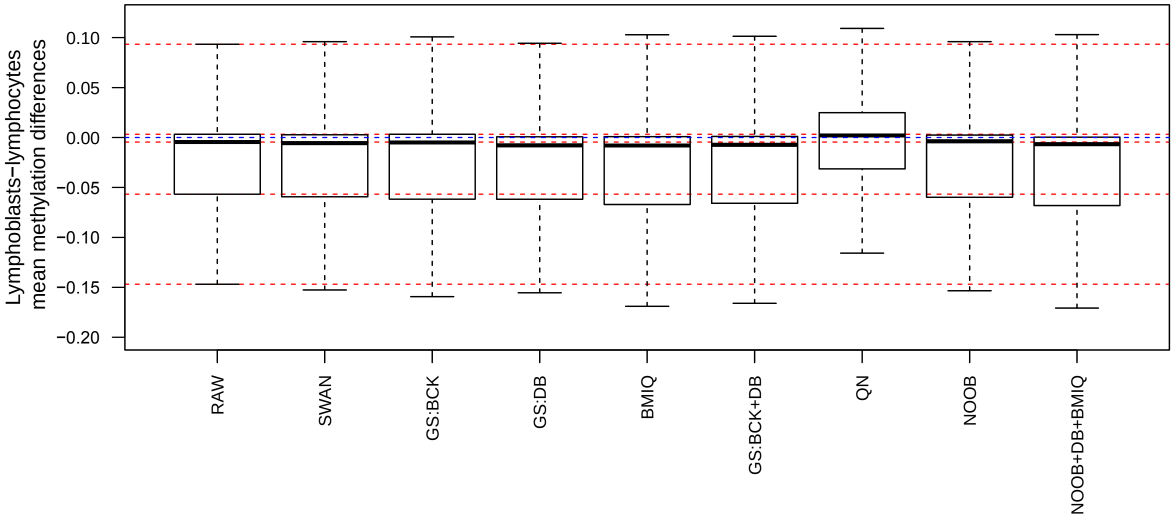

Supplementary Figure 8: Distribution of mean methylation differences between LCL samples and lymphocyte samples, accoring to data transformation method. Boxplot values for the raw data are extended with red horizontal dashed lines. Blue dashed line at 0. Outliers are not plotted.

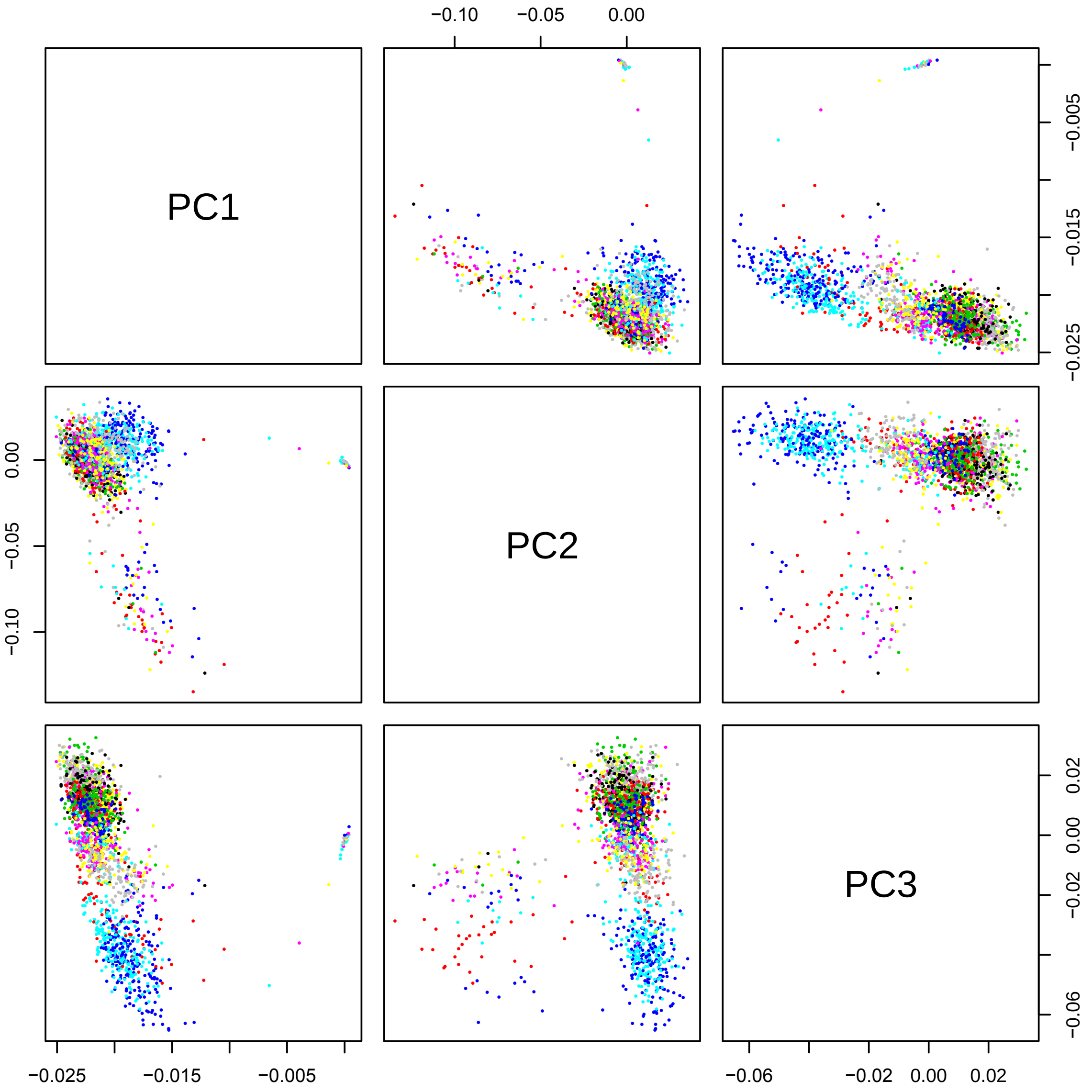

Supplementary Figure 9: Principal component analysis of 2228 samples. The first 3 principal components are plotted; collectively they explain 95% of the total variance. Each sample is color coded according to its 96-well DNA plate.

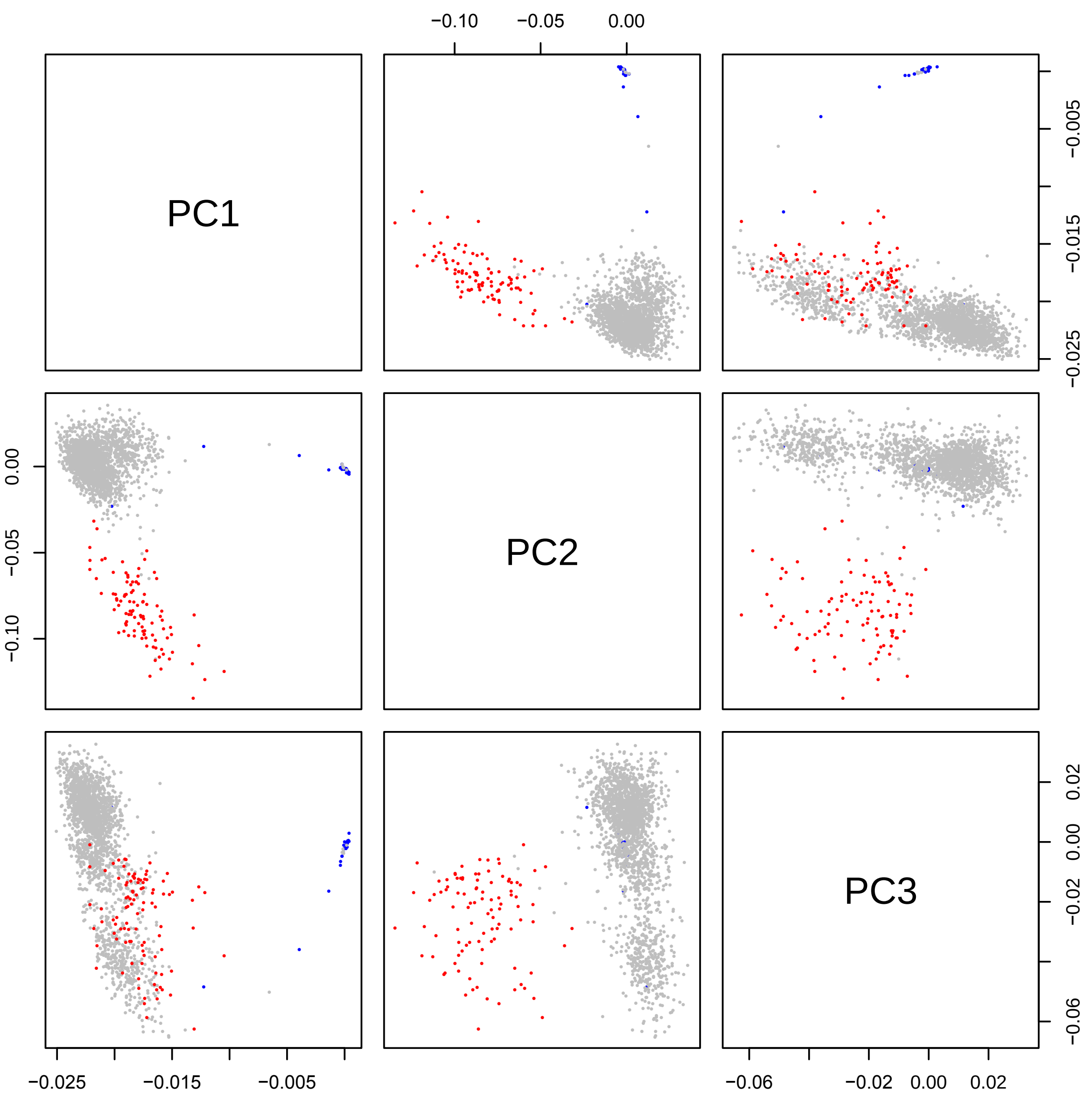

Supplementary Figure 10: Principal component analysis of 2228 samples. The first 3 principal components are plotted; collectively they explain 95% of the total variance. Each sample is color coded according to the source of its DNA: from lymphocytes (gray) or lymphoblastoid cell lines (red); blue points represent spots left blank on the arrays.

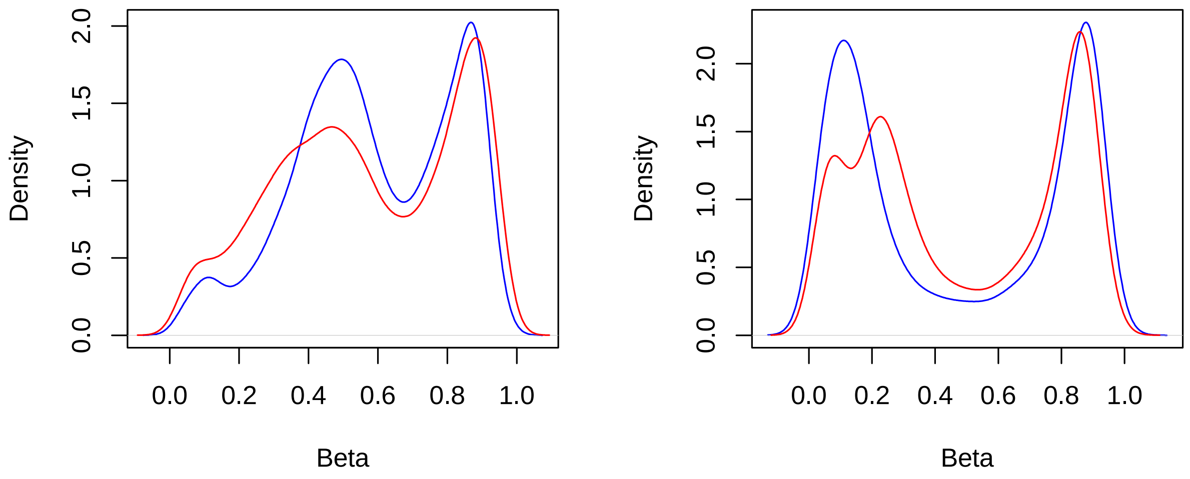

Supplementary Figure 11: Densities of X chromosome methylation values (Beta) in females (left) and males (right). Blue: raw data; red: quantile normalized data.

Supplementary Methods

Normalization of methylation values

Red and green intensities -- or the methylation values calculated from them -- were normalized (1) using the algorithms described in the *GenomeStudio Methylation Module v1.8 User Guide* (Illumina, Inc.); these algorithms use control probes specifically designed to normalize the intensities (between sample color bias adjustment) and estimate the level of background intensities, which can be subtracted from signal intensities; (2) using the same color adjustment probes and algorithms, but using out-of-band signals (from the Infinium I probes) to estimate and correct for background intensities (NOOB) [1]; (3) using SWAN [2] and (4) BMIQ [3], two methods that adjust the differences in the distributions of methylation values between Infinium I and Infinium II probes; (5) quantile normalization [4] on red and green intensities separately. We illustrate the adverse effects of quantile normalization on methylation values by comparing DNA from samples that were extracted from lymphocytes with 99 DNAs extracted from lymphoblastoid cell lines (LCL); these two groups are expected to display substantially different methylation profiles throughout the genome [5]. This situation does not warrant the use of normalization methods that assume similar intensity distributions or similar biological conditions in all samples [6].

For each normalization strategy, we compared the resulting β values in all pairs of duplicate samples. Sixty-five SNPs with high heterozygosity were assayed on the HumanMethylation450 array; they were used to ensure that duplicates were indeed corresponding to the same DNA sample. We counted the number of sites for which the β values differed by more than 1%, 2%, 5% and 10% in pairs of duplicates, either duplicates belonging to the same 96-well DNA plate (intra-plate pairs) or different plates (inter-plate pairs). We further stratified these counts in

strata of CpG sites based on their average β values, calculated from the raw methylation profiles in all samples. Supplementary Fig. 4 illustrates these counts, averaged over all intra-plate duplicate pairs, while Supplementary Fig. 5 illustrates counts averaged over inter-plate duplicate pairs. Based on these, the NOOB background correction consistently outperforms GenomeStudio's, while BMIQ peak-based correction globally outperforms SWAN, but not consistently throughout all ranges of methylation profiles. Quantile normalization (QN) underperforms globally compared to NOOB, but not consistently. Combining NOOB with *GenomeStudio's* description of dye-bias correction, followed by BMIQ peak-based correction outperforms all methods taken individually, but not consistently. For this combination, the differences between the beta values in duplicates are markedly reduced, except for intermediate methylation values (20-80% range). Supplementary Fig. 6 and 7 in focus on the correlation coefficients between the set of beta values in pairs of duplicates; similar conclusions can be drawn.

GenomeStudio's implementation of background and dye-bias correction (GS:BCK+DB) performs generally well and is a reasonable choice. This is a somewhat discrepant message compared to the interpretation found in [7]. In there, it is argued that using GenomeStudio's background and dye-bias corrections leads to increased differences between duplicates compared to raw data. However, their interpretation was based on comparing the M-values in duplicates instead of beta values. Since M is a logit transformation of β ($M=\log_2\{\beta/(1-\beta)\}$), M becomes larger as beta approaches 0 or 1; small differences in the β scale translate into larger differences in the M scale. Since applying a background correction moves β values closer to these boundaries, the associated M-values get larger and differences are amplified. To illustrate, the

difference between the M-values corresponding to beta values of 8% (M=-3.52) and 4% (-4.58) is approximately the same as the difference between M-values corresponding to beta values of 0.2% (-8.96) and 0.1% (-9.96). We do not think that the M scale is an appropriate scale to compare methods, especially if background correction is involved.

Despite words of warnings against its use [6], quantile normalization has been recommended as a normalization method of choice in the literature [7]. While quantile normalization appears to improve reproducibility, subsets of samples with substantial differences in their methylation profiles will see substantial changes following quantile normalization, and will look more alike. Supplementary Fig. 8 illustrates this, where we compare the mean differences in methylation values between samples whose DNA were extracted from lymphocytes and from LCLs. LCL samples (which were removed in the SNP-CpG association analyses) display substantially different methylation profiles throughout the genome compared to lymphocytes [5] (Supplementary Fig. 9-10): globally, CpG sites tend to display lower methylation levels in LCL samples compared to lymphocyte samples at a majority of sites (Supplementary Fig. 8); this observation is preserved irrespective of the data transformation method (between 63-72% of sites display lower methylation in LCL, depending on the data transformation method), except for quantile normalization where the differences are more symmetrical and interpretation is qualitatively different (45% of sites display lower methylation values in LCL and skewness is noticeably reduced). While it could be argued that the possibility exists that quantile normalization is the only method that appropriately transforms the data (in absence of a gold standard dataset), we note that the mean methylation values were adjusted for sex, age, array and position on the array, and were calculated only for arrays presenting at least two LCL and two

lymphocyte samples; batch, technical or other effects are thus not expected to have a strong influence on those adjusted mean differences. In absence of a gold standard dataset, extracting probes from chromosome X and applying a data transformation method only on them can be used as a model to confirm that the method does not disrupt the unique features of male and female methylation profiles. Females typically show 3 methylation peaks (a methylated peak, an unmethylated peak and, because of X inactivation, a hemi-methylated peak) while males only display two peaks (a methylated peak and an unmethylated one). Supplementary Fig. 11 is a typical illustration of the effects of quantile normalization on X chromosome probes: after normalization, males show 3 peaks, similar to females, while in females the weight of the hemi-methylated peak moves toward the unmethylated state.

Supplementary References

1. Triche TJ Jr, Weisenberger DJ, Van Den Berg D, Laird PW, Siegmund KD: **Low-level processing of Illumina Infinium DNA Methylation BeadArrays.** *Nucleic Acids Res*. 2013, **41**:e90.Maksimovic J, Gordon L, Oshlack A. **SWAN: Subset-quantile within array normalization for illumina infinium HumanMethylation450 BeadChips.** *Genome Biol.* 2012, 13:R44.
2. Teschendorff AE, Marabita F, Lechner M, Bartlett T, Tegner J, Gomez-Cabrero D, Beck S. **A beta-mixture quantile normalization method for correcting probe design bias in Illumina Infinium 450 k DNA methylation data.** *Bioinformatics*. 2013, **29**:189-196.
3. Bolstad BM, Irizarry RA, Astrand M, Speed TP: **A comparison of normalization methods for high density oligonucleotide array data based on variance and bias.** *Bioinformatics* 2003, **19**:185-193.
4. Grafodatskaya D, Choufani S, Ferreira JC, Butcher DT, Lou Y, Zhao C, Scherer SW, Weksberg R: **EBV transformation and cell culturing destabilizes DNA methylation in human lymphoblastoid cell lines.** *Genomics* 2010, **95**:73-83.
5. Laird PW: **Principles and challenges of genomewide DNA methylation analysis.** *Nat Rev Genet* 2010, **11**:191-203.
6. Marabita F, Almgren M, Lindholm ME, Ruhrmann S, Fagerström-Billai F, Jagodic M, Sundberg CJ, Ekström TJ, Teschendorff AE, Tegnér J, Gomez-Cabrero D. **An evaluation of analysis pipelines for DNA methylation profiling using the Illumina HumanMethylation450 BeadChip platform.** *Epigenetics*. 2013, **8**:333-346.
